# The PAF Complex and Prf1/Rtf1 Delineate Distinct Cdk9-Dependent Pathways Regulating Transcription Elongation in Fission Yeast

**DOI:** 10.1371/journal.pgen.1004029

**Published:** 2013-12-26

**Authors:** Jean Mbogning, Stephen Nagy, Viviane Pagé, Beate Schwer, Stewart Shuman, Robert P. Fisher, Jason C. Tanny

**Affiliations:** 1 Department of Pharmacology and Therapeutics, McGill University, Montreal, Canada; 2 Department of Microbiology and Immunology, Weill Cornell Medical College, New York, New York, United States of America; 3 Molecular Biology Program, Memorial Sloan-Kettering Cancer Center, New York, New York, United States of America; 4 Department of Structural and Chemical Biology, Icahn School of Medicine at Mount Sinai, New York, New York, United States of America; University of California San Francisco, United States of America

## Abstract

Cyclin-dependent kinase 9 (Cdk9) promotes elongation by RNA polymerase II (RNAPII), mRNA processing, and co-transcriptional histone modification. Cdk9 phosphorylates multiple targets, including the conserved RNAPII elongation factor Spt5 and RNAPII itself, but how these different modifications mediate Cdk9 functions is not known. Here we describe two Cdk9-dependent pathways in the fission yeast *Schizosaccharomyces pombe* that involve distinct targets and elicit distinct biological outcomes. Phosphorylation of Spt5 by Cdk9 creates a direct binding site for Prf1/Rtf1, a transcription regulator with functional and physical links to the Polymerase Associated Factor (PAF) complex. PAF association with chromatin is also dependent on Cdk9 but involves alternate phosphoacceptor targets. Prf1 and PAF are biochemically separate in cell extracts, and genetic analyses show that Prf1 and PAF are functionally distinct and exert opposing effects on the RNAPII elongation complex. We propose that this opposition constitutes a Cdk9 auto-regulatory mechanism, such that a positive effect on elongation, driven by the PAF pathway, is kept in check by a negative effect of Prf1/Rtf1 and downstream mono-ubiquitylation of histone H2B. Thus, optimal RNAPII elongation may require balanced action of functionally distinct Cdk9 pathways.

## Author Summary

The expression of many eukaryotic genes is regulated during the elongation phase of transcription. Cyclin-dependent kinase 9 (CDK9) enzymes are key positive regulators of RNA polymerase II (RNAPII) elongation, and are required for co-transcriptional events such as mRNA processing and chromatin modification. Cdk9 phosphorylates RNAPII and the elongation factor Spt5, but the molecular pathways that underlie Cdk9 functions in transcription remain poorly defined. Using fission yeast *Schizosaccharomyces pombe* as a model system, we have identified two Cdk9-dependent pathways that modulate RNAPII elongation. One pathway involves the direct interaction of phosphorylated Spt5 with Prf1/Rtf1, a multi-functional transcriptional regulator. A second pathway, involving a different Cdk9 target, directs the Polymerase Associated Factor (PAF) complex to transcribed chromatin. The Prf1 and PAF pathways are functionally distinct and have opposing effects on the RNAPII elongation complex. Our data provide novel insights into Cdk9 functions and suggest that Cdk9 auto-regulatory mechanisms are an important feature of RNAPII elongation.

## Introduction

Regulation of RNA polymerase II (RNAPII) transcription elongation affects the expression of many eukaryotic genes [1]. A central regulator of metazoan elongation is positive transcription elongation factor b (P-TEFb), a complex comprising cyclin-dependent kinase 9 (CDK9) and a cyclin partner. P-TEFb was identified on the basis of its biochemical activity in overcoming promoter-proximal RNAPII pausing imposed by DRB-sensitivity inducing factor (DSIF; a complex of Spt4 and Spt5) and a negative elongation factor (NELF) [2]–[5]. Cdk9 orthologs are present and essential in *Saccharomyces cerevisiae* (Bur1) and *Schizosaccharomyces pombe* (Cdk9), where they have imputed positive roles in RNAPII elongation [6]–[12]. Cdk9 phosphorylates multiple proteins involved in elongation, including Spt5 and the Rpb1 subunit of RNAPII [13]. In addition to regulating elongation, these phosphorylations are implicated in the recruitment of chromatin-modifying enzymes and mRNA 3′-end processing factors [11], [14]–[16]. Cdk9 can also phosphorylate and activate the histone H2B ubiquitin-conjugating enzyme RAD6, indicating multiple links between Cdk9 and chromatin modification [17], [18].

Cdk9 phosphorylates both Rpb1 and Spt5 within repeated amino acid motifs at their C-termini. The Rpb1 C-terminal domain (CTD) repeat has consensus sequence Y^1^SPTSPS^7^ and is involved in coordinating co-transcriptional events [14]. Cdk9 can phosphorylate multiple sites within the CTD heptad, but its roles in RNAPII elongation have been commonly attributed to Ser2 phosphorylation [13]. Spt5 contains a C-terminal repeat domain (which varies in size and sequence among taxa) that is phosphorylated by Cdk9 on a conserved threonine residue within the repeated motif [4], [11], [16], [19]. The relative contributions of specific Cdk9 targets to its various functions in transcription are not known.

The Polymerase-Associated Factor (PAF) complex is a key mediator of Cdk9 function during elongation. PAF is minimally composed of conserved subunits Paf1, Leo1, Cdc73, and Ctr9 [20], and its functions broadly overlap with those of Cdk9. In vitro, PAF cooperates with the phosphorylated Spt5 CTD to relieve promoter-proximal RNAPII pausing, and stimulates RNAPII elongation on DNA and chromatin templates [21]–[24]. Like Cdk9, PAF also functions in mRNA 3′ end processing and co-transcriptional chromatin modification [25]–[28]. In budding yeast, PAF recruitment to transcribed genes requires Bur1-dependent Spt5 phosphorylation, which promotes a direct interaction between the Spt5 CTD and the Rtf1 subunit of PAF [15], [16], [18], [29]–[31].

Rtf1 is a stable component of PAF in budding yeast, but is biochemically separate from PAF in metazoans [22], [26], [32]–[37]. Both PAF and Rtf1 are present at transcribed genes and are required for co-transcriptional formation of mono-ubiquitylated histone H2B (H2Bub1) and methylation of histone H3 lysine 4 (H3K4me) [25], [26]. Mutation or knockdown of *PAF1* and *RTF1* revealed shared functions in telomeric gene silencing in *S. cerevisiae*, somite development and hematopoiesis in zebrafish, regulation of flowering time in *A. thaliana*, and mouse embryonic stem cell pluripotency [25], [38]–[41]. In contrast, zebrafish Rtf1 and PAF were found to have distinct functions in cardiac development [34]. Characterization of PAF mutants in *S. cerevisiae* revealed pleiotropic deficiencies for *paf1Δ* and *ctr9Δ* mutants but few phenotypes overall in an *rtf1Δ* strain [42]. Indeed, an *rtf1Δ* mutation was found to suppress the phenotypes of *paf1Δ*, suggesting that the relationship between Rtf1 and PAF could, in some circumstances, be antagonistic [37]. Lethality of *bur1Δ* in budding yeast is also suppressed by *rtf1Δ*, as are phenotypes caused by mutation of the associated cyclin Bur2 [43], [44]. Thus, Rtf1 may counteract an important shared function of Cdk9 and PAF.

Although both Rtf1 and PAF are required for formation of H2Bub1 and H3K4me, genetic analyses in *S. cerevisiae* indicate a particularly close functional connection between Rtf1 and these histone modifications [45], [46]. While the precise gene regulatory functions of H2Bub1 and H3K4me have not been elucidated, H2Bub1, like Rtf1, has been found to oppose Cdk9. In the fission yeast *S. pombe*, phenotypes associated with loss of H2Bub1 are suppressed by reduction of activity of Cdk9. These include a general 3′ shift of RNAPII density within genes, a pattern that could reflect enhanced elongation through coding regions caused by aberrant Cdk9 function [11]. H2Bub1 itself depends upon Cdk9 activity toward Spt5, and also contributes positively to Spt5 phosphorylation (Spt5-P) levels. Thus, H2Bub1 and Spt5-P may be part of an auto-regulatory mechanism that fine-tunes Cdk9 functions. The Cdk9 function antagonized by H2Bub1 involves a target other than Spt5, suggesting the involvement of a distinct Cdk9 pathway [11].

Using the fission yeast *S. pombe* as a model system, we report that PAF and Rtf1 (which we designate Prf1 in *S. pombe*) are involved in two distinct and opposing regulatory pathways, both of which depend on Cdk9 activity. We further show that these divergent functions involve different Cdk9 targets, and mirror the opposing functions of Cdk9 and H2Bub1 in elongation.

## Results

### PAF and Prf1/Rtf1 are biochemically separate and functionally distinct

**Figure 1:**
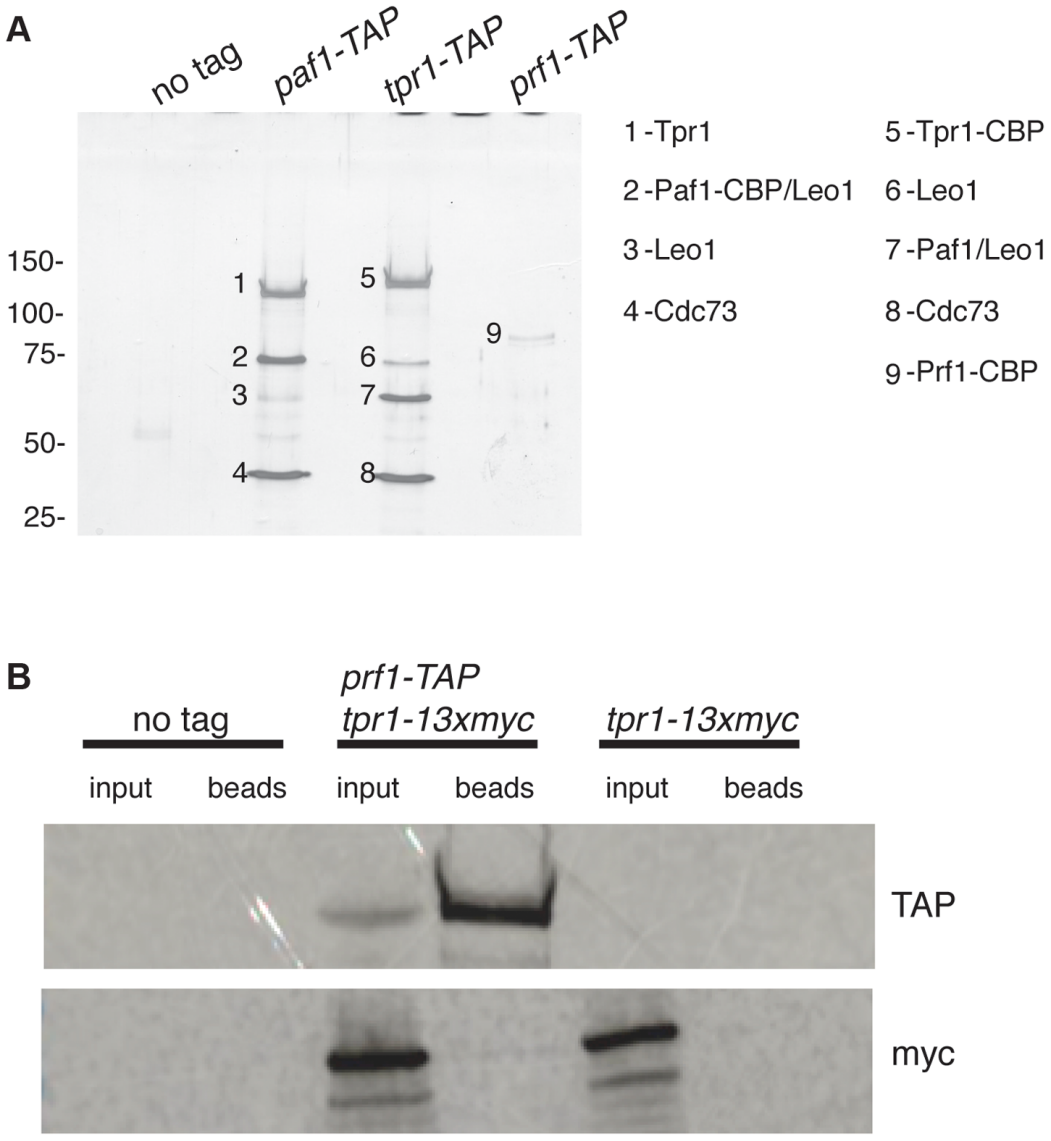
Prf1 does not stably associate with the PAF complex. (**A**) TAP purification was carried out using whole cell extracts from the indicated strains and purified material was analyzed by SDS-PAGE and silver staining. The proteins contained in the labeled bands were identified by mass spectrometry (as indicated on the right). “CBP” denotes the residual fusion to the calmodulin binding peptide resulting from the TAP procedure. Leo1 was detected in two bands that also contained either Paf1-CBP (in the *paf1-TAP* lane) or Paf1 (in the *tpr1-TAP* lane). Molecular weight standards (in kD) are denoted on the left. (**B**) Single-step TAP purifications were performed using extracts from the indicated strains. 5% of input fractions (“input”) and 50% of bead-bound fractions (“beads”) were analyzed by SDS-PAGE and western blotting.

To further explore the relationship between Cdk9 activity and H2Bub1 in *S. pombe*, we assessed the biochemical and functional properties of orthologs of known PAF complex subunits. We first tagged the genes encoding homologs of Paf1 (systematic ID # SPAC664.03), Ctr9 (SPAC27D7.14c; named *tpr1* ^+^ in *S. pombe*), and Rtf1 [SPBC651.09c; which we henceforth refer to as Prf1, for PAF-related factor 1, to avoid confusion with the unrelated gene SPAC22F8.07c currently annotated as *rtf1* ^+^ [47]] with the tandem affinity purification (TAP) tag and purified the factors from whole-cell extracts [48]. Purified material was analyzed by SDS-PAGE alongside that from an untagged strain. Polypeptides uniquely present in the TAP purifications were excised from the SDS gel and analyzed by tandem mass spectrometry. Material purified from *paf1-TAP* and *tpr1-TAP* strains contained orthologs of the previously defined PAF complex components Paf1, Leo1 (SPBC13E7.08c), Tpr1, and Cdc73 (SPBC17G9.02c) (Figure 1A). Prf1 was not visible by SDS-PAGE in the Tpr1-TAP or Paf1-TAP preparations, nor were PAF components visible in the Prf1-TAP preparation (Figure 1A). Thus, Prf1 and PAF are predominantly separate in *S. pombe* cell extracts.

To further confirm this finding we carried out in-solution mass spectrometry analysis on the Tpr1-TAP and Prf1-TAP preparations. Material derived from an untagged strain was analyzed in parallel as a control. This analysis identified the core PAF subunits Paf1, Leo1, Tpr1, and Cdc73 with high confidence in the Tpr1-TAP preparation, but not in the control or Prf1-TAP preparations (see Table S1). Conversely, Prf1 was identified with high confidence only in the Prf1-TAP purification.

We also investigated whether we could detect association between Prf1 and PAF in a single-step isolation procedure. We constructed a strain carrying both *prf1-TAP* and *tpr1-13xmyc* fusion genes. Whole-cell extracts from this strain, as well as from a *tpr1-13xmyc* strain, were incubated with IgG-sepharose beads to isolate Prf1-TAP and purified material was analyzed by western blotting. Whereas Prf1-TAP was efficiently purified, no Tpr1-13xmyc protein could be detected, further arguing that Prf1 and PAF do not stably associate in *S. pombe* cell extracts (Figure 1B).

**Figure 2:**
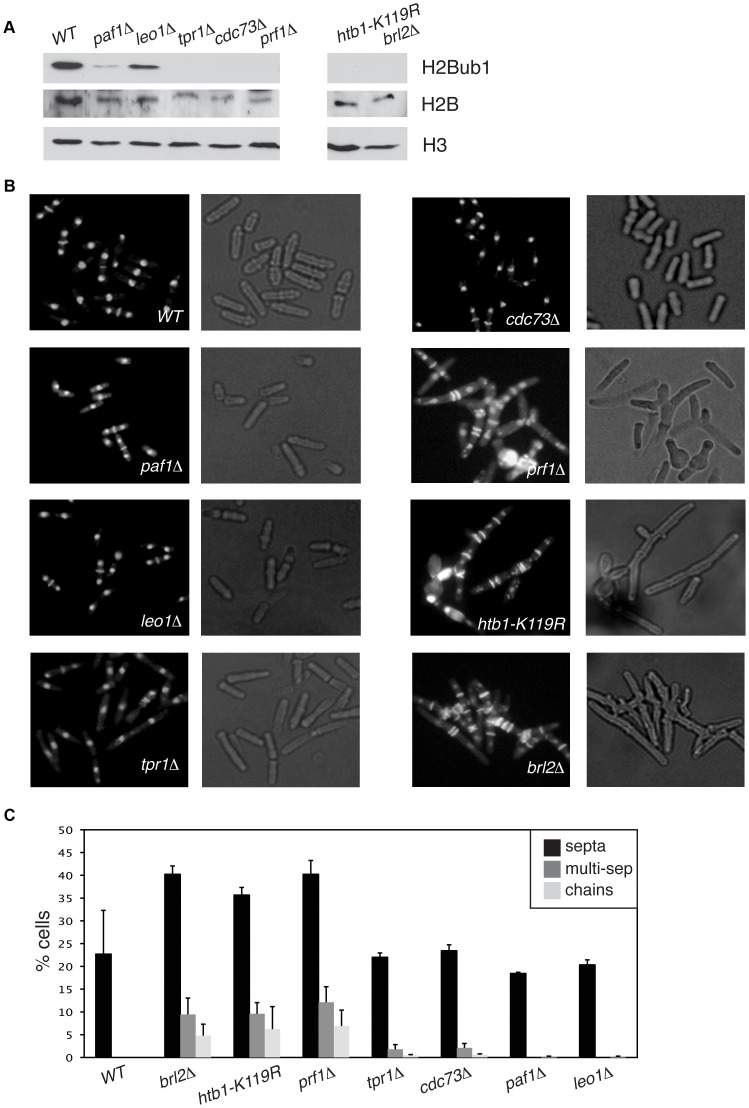
Prf1 and PAF have shared roles in histone H2B ubiquitylation but are functionally distinct. (**A**) Whole-cell extracts from strains of the indicated genotypes were analyzed by SDS-PAGE and western blotting with the indicated antibodies. (**B**) Cells of the indicated genotypes were fixed, stained with DAPI/calcofluor, and visualized by microscopy. For each strain fluorescent images (denoting DAPI/calcofluor staining) are shown on the left; bright-field images are shown on the right. (**C**) Quantification of the abnormal septation patterns in the indicated strains. “Septa” refers to cells with at least one visible division septum, “multi-sep” refers to cells with twinned septa or multiple septa between two nuclei, and “chains” refers to unseparated chains of cells. Error bars denote standard deviations from 3 independent experiments.

We found that deletion of the *S. pombe prf1* ^+^ gene, or of the *tpr1* ^+^ and *cdc73* ^+^ genes encoding PAF components, resulted in a total loss of H2Bub1 as gauged by immunoblotting, comparable to that observed in *htb1-K119R* (ubiquitin acceptor site) or *brl2*Δ (E3 ubiquitin ligase) mutants (Figure 2A). The presence of normal levels of H2Bub1 in the *prf1-TAP* and *tpr1-TAP* strains affirmed the functionality of the respective TAP-tagged proteins (Figure S1). As expected from the established dependence of H3K4me on H2Bub1 in yeast [49], [50], the *prf1Δ*, *tpr1Δ*, and *cdc73Δ* mutations also strongly diminished tri- and dimethyl forms of H3K4me, albeit to varying extents (Figure S2). Thus, Prf1 and PAF have a shared function in co-transcriptional histone modification. The *paf1*Δ and *leo1*Δ mutations caused only partial reduction in H2Bub1 and H3K4me levels, indicating divergent roles of PAF complex subunits in promoting these modifications (Figures 2A and S2). Because the H3K36me modification was maintained in these mutants, we infer a specific role for *S. pombe* PAF and Prf1 in the H2Bub1/H3K4me chromatin modification axis (Figure S2).

Loss of H2Bub1 caused by *htb1-K119R* or *brl2*Δ mutations is associated with cell morphology and septation defects (including “twinned” septa and unseparated chains of cells) [11]. Microscopic examination of DAPI/calcofluor-stained cells revealed that *prf1*Δ caused qualitatively and quantitatively similar phenotypes (Figures 2B and 2C). To our surprise, the PAF complex mutations *tpr1*Δ and *cdc73*Δ, which eliminated H2Bub1 and reduced H3K4me levels, caused minimal cell morphology and septation defects. This indicates functional divergence between PAF and other factors promoting H2Bub1.

**Figure 3:**
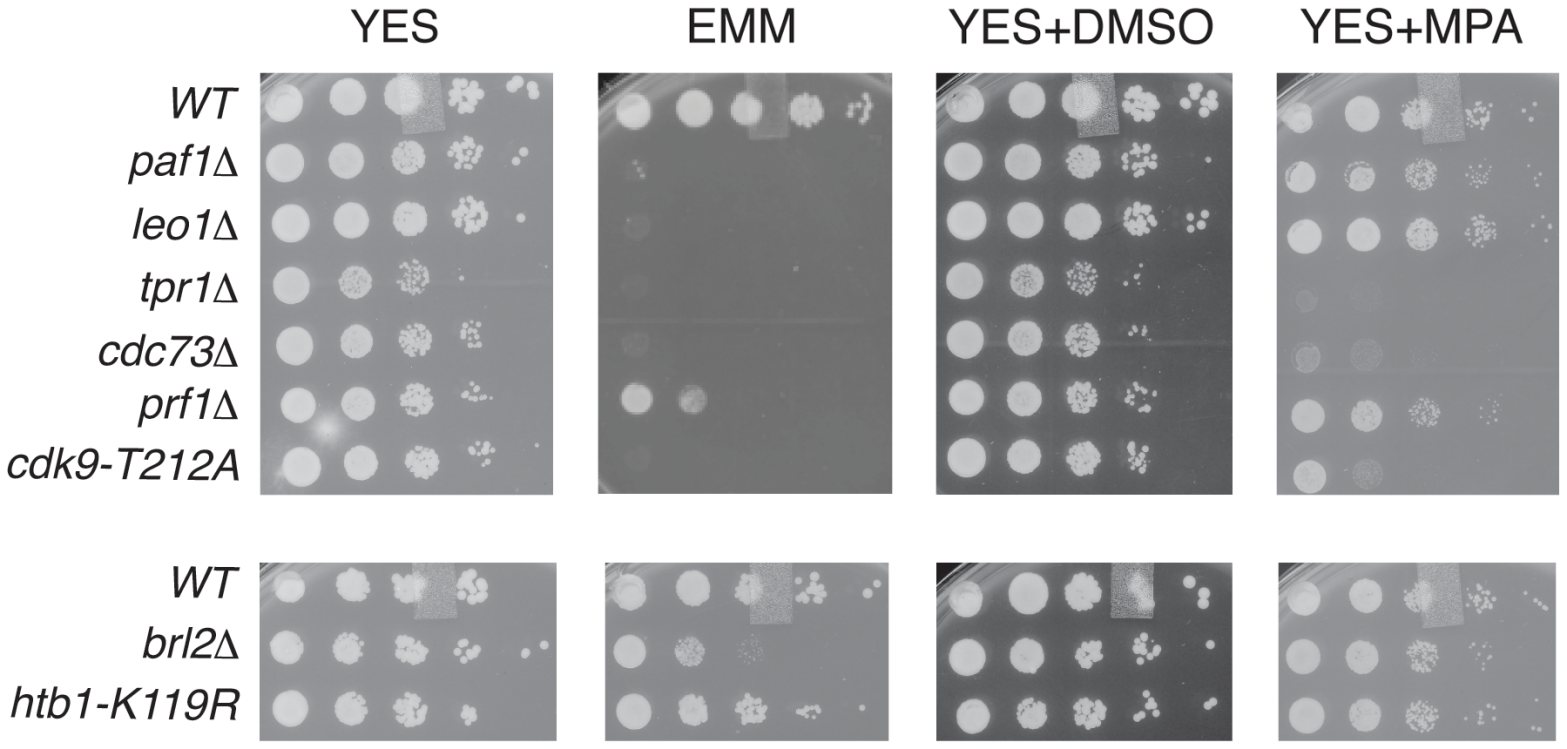
PAF mutant phenotypes resemble *cdk9* loss-of-function. Five-fold serial dilutions of the indicated strains were spotted on agar plates containing rich media (YES), minimal media (EMM), rich media with dimethyl sulfoxide (YES+DMSO), or rich media with mycophenolic acid (YES+MPA).

To further probe the functional relationships between Cdk9, PAF, Prf1, and H2Bub1, we assessed growth of the relevant mutant strains under conditions previously shown to make requirement for Cdk9 activity more stringent, including minimal medium (EMM) and medium containing the nucleoside biosynthesis inhibitor mycophenolic acid (MPA) [51]. We found that *tpr1*Δ and *cdc73*Δ phenocopied the hypomorphic *cdk9-T212A* mutant (which lacks the phosphorylation site for the CDK-activating enzyme Csk1; [7]) under these conditions, arguing for a functional link between these factors and Cdk9 activity (Figure 3). The *paf1*Δ and *leo1*Δ mutants shared the dramatic EMM sensitivity of *tpr1*Δ and *cdc73*Δ but were only mildly sensitive to MPA. Importantly, *htb1-K119R* and *brl2*Δ mutants lacked the phenotypic signature associated with reduced Cdk9 activity and loss of PAF, as did *prf1*Δ (Figure 3). The *brl2*Δ and *prf1*Δ mutations did cause moderate growth defects on EMM, perhaps due to H2Bub1-independent functions of Brl2 and Prf1 proteins. Thus, whereas PAF is functionally aligned with Cdk9, Prf1 is intimately connected to factors directly involved in H2Bub1 formation.

### Recruitment of PAF and Prf1 to transcribed chromatin involves distinct Cdk9 targets

**Figure 4:**
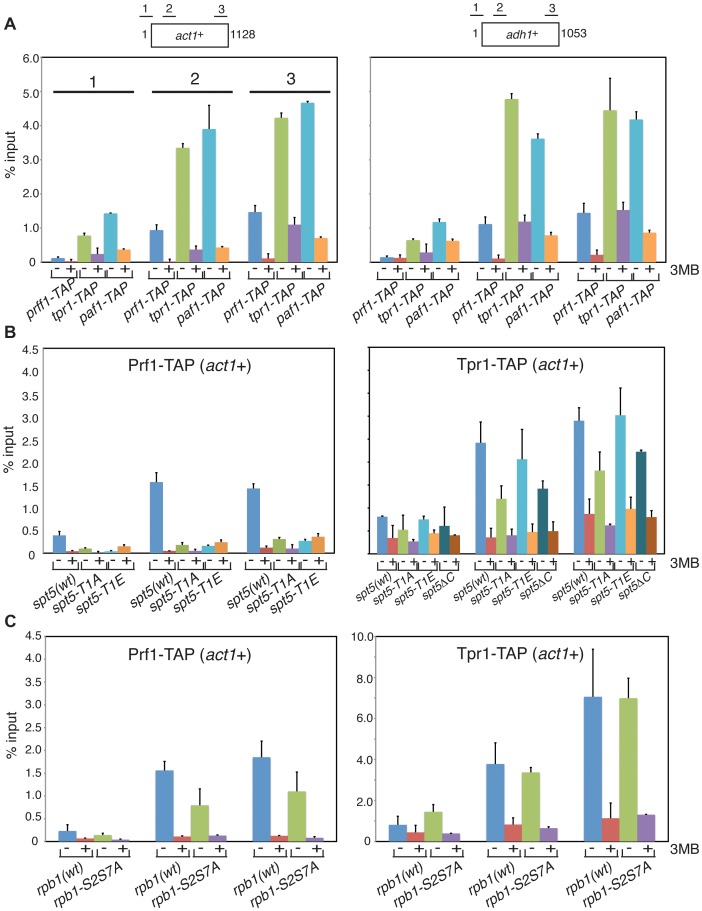
Prf1 and PAF are recruited to chromatin via alternate mechanisms. (**A**) Strains used for ChIP with IgG resin (recognizing the TAP tag) are indicated at the bottom. All strains also harbored the *cdk9^as^* allele and were treated with either DMSO (−) or 20 µM 3-MB-PP1 (+) for two hours before crosslinking for ChIP (also indicated by “3MB” at the far right). Assays were quantified by qPCR using primers specific for the *act1* ^+^ (left) or *adh1* ^+^ (right) genes. Lengths of the gene coding regions (in base pairs) and positions of PCR amplicons are indicated at the top. Numbering of the datasets for the corresponding PCR amplicons shown for *act1* ^+^ (left) is used throughout the figure. Error bars denote standard deviations from 2–3 independent experiments. (**B and C**) Strains of genotypes indicated at the bottom were used for ChIP of either Prf1-TAP (left) or Tpr1-TAP (right). Cultures were treated with DMSO or 3-MB-PP1 as in (A).

To further define the distinct functions of PAF and Prf1 we investigated mechanisms that target these factors to transcribed genes. Prompted by findings in budding yeast linking PAF chromatin association to Bur1 activity [15], [16], [30], we performed chromatin immunoprecipitation (ChIP) in *prf1-TAP*, *tpr1-TAP*, and *paf1-TAP* strains that also carried a Cdk9 variant (*cdk9^as^*) engineered to be sensitive to inhibition by bulky ATP analogues [12]. We found that chromatin recruitment of all three proteins to the constitutive *act1* ^+^ and *adh1* ^+^ genes depended on Cdk9 activity (Figure 4A). Acute inhibition of Cdk9^as^ in these strains had no effect on overall levels of the respective TAP-tagged proteins (Figure S3A) and did not greatly impair RNAPII occupancy at *act1* ^+^ or *adh1* ^+^ (Figure S3B). This argues that chromatin association of both PAF and Prf1 depends on Cdk9 activity.

We reasoned that different Cdk9 targets might mediate the distinct functions of PAF and Prf1. The Spt5 CTD is a specific target of Cdk9 in *S. pombe*; the Spt5 CTD consists of tandem copies of a nonapeptide motif of consensus sequence T^1^PAWNSGSK^9^. We reported previously that changing the Thr1 phosphoacceptor site to alanine reduced H2Bub1 levels [11]. We analyzed Prf1 and PAF recruitment to transcribed chromatin by ChIP in strains harboring Spt5 CTD mutations. The strains were constructed by replacing the endogenous *spt5* ^+^ CTD domain (which contains 18 nonapeptide repeats) with a truncated (but fully functional) CTD domain containing eight consensus repeats or otherwise identical repeat arrays with T1A or T1E mutations [19]. The strains also carried the *cdk9^as^* allele, which allowed assessment of the contribution of specific loss of Spt5-P to the effect of general Cdk9^as^ inhibition.

The *spt5-T1A* mutation reduced chromatin association of Prf1 at *act1* ^+^ and *adh1* ^+^ to a level close to that observed upon inhibition of Cdk9^as^ (5- to 10-fold; Figures 4B and S4A). Thus, Prf1 recruitment to transcribed chromatin strongly depends on phosphorylation of the Spt5 CTD by Cdk9. The phosphomimetic *spt5-T1E* mutation also impaired ChIP of Prf1, but rendered the residual crosslinking of Prf1 Cdk9-independent. Thus, although a negative charge at all Thr1 positions within a truncated CTD is not sufficient for normal Prf1 recruitment it does allow some Prf1 binding through an altered, Cdk9-independent mechanism.

In contrast, ChIP of Tpr1-TAP revealed Cdk9-dependent chromatin association in the presence of *spt5-T1A* and *spt5-T1E* mutations, as well as in a strain in which the Spt5 CTD is deleted altogether (*spt5ΔC*)(Figures 4B and S4A). This argues that PAF recruitment to chromatin involves a Cdk9 target other than the Spt5 CTD. The modest (∼2-fold) decrease in ChIP of Tpr1-TAP in the *spt5-T1A* and *spt5ΔC* mutants (that was not apparent in the phosphomimetic *spt5-T1E* mutant) suggests that Spt5-P does make some contribution to PAF recruitment. These data point to the existence of two functionally distinct Cdk9 pathways (which we will refer to as the PAF pathway and the Prf1 pathway) that involve distinct phosphoacceptor targets.

We note that global levels of Spt5-P were reduced by *prf1*Δ, as was found previously for *htb1-K119R* and *brl2*Δ mutations (Figure S5A). This implicates Prf1 as part of the positive feedback loop linking Spt5-P and H2Bub1. Mutations in PAF components either did not affect (*tpr1*Δ and *cdc73*Δ) or increased (*paf1*Δ and *leo1*Δ) global levels of Spt5-P, further highlighting the specific functional connection between Spt5-P and the Prf1 pathway.

The Rpb1 CTD is a target of Cdk9. In *S. pombe*, Cdk9 contributes to global levels of Rpb1-Ser2 phosphorylation and promotes the formation of multiply phosphorylated Rpb1 CTD repeats [12], [51]. Budding yeast PAF components Cdc73 and Ctr9 both interact with phosphorylated Rpb1 CTD peptides in vitro and enhance global levels of Rpb1-Ser2 phosphorylation in vivo [30], [32], [52]. This latter function is shared by the corresponding PAF components in *S. pombe* (but not by Prf1; see Figure S5B). To investigate the role of Rpb1 CTD phosphorylation in Prf1 and PAF recruitment, we carried out ChIP assays in strains harboring phosphoacceptor site mutations in all the Rpb1 CTD repeats. The “wild-type” control in these experiments carried a truncated but fully functional Rpb1 CTD domain [53]. ChIP assays using an *rpb1-S2AS7A* double mutant (in which both serines 2 and 7 are changed to alanine in every CTD repeat) showed very little effect on chromatin association of either Prf1-TAP or Tpr1-TAP at the active genes analyzed (although a ∼2-fold, gene-specific reduction of Prf1-TAP association was observed at *act1* ^+^) (Figures 4C and S4B).

We also tested the effect of an *rpb1-S5A* mutation, the lethality of which was bypassed by fusion of *rpb1-S5A* to the *MCE1* gene encoding the mammalian mRNA capping enzyme [53]. As compared to the wild-type *rpb1-MCE1* fusion, the *rpb1-S5A-MCE1* fusion had little impact on crosslinking of either Prf1 or Tpr1 to the two loci tested (Figure S6). These data suggest that Rpb1 CTD phosphorylation on serines 2, 5, or 7 is not required for Prf1 or PAF association with transcribed chromatin in vivo.

**Figure 5:**
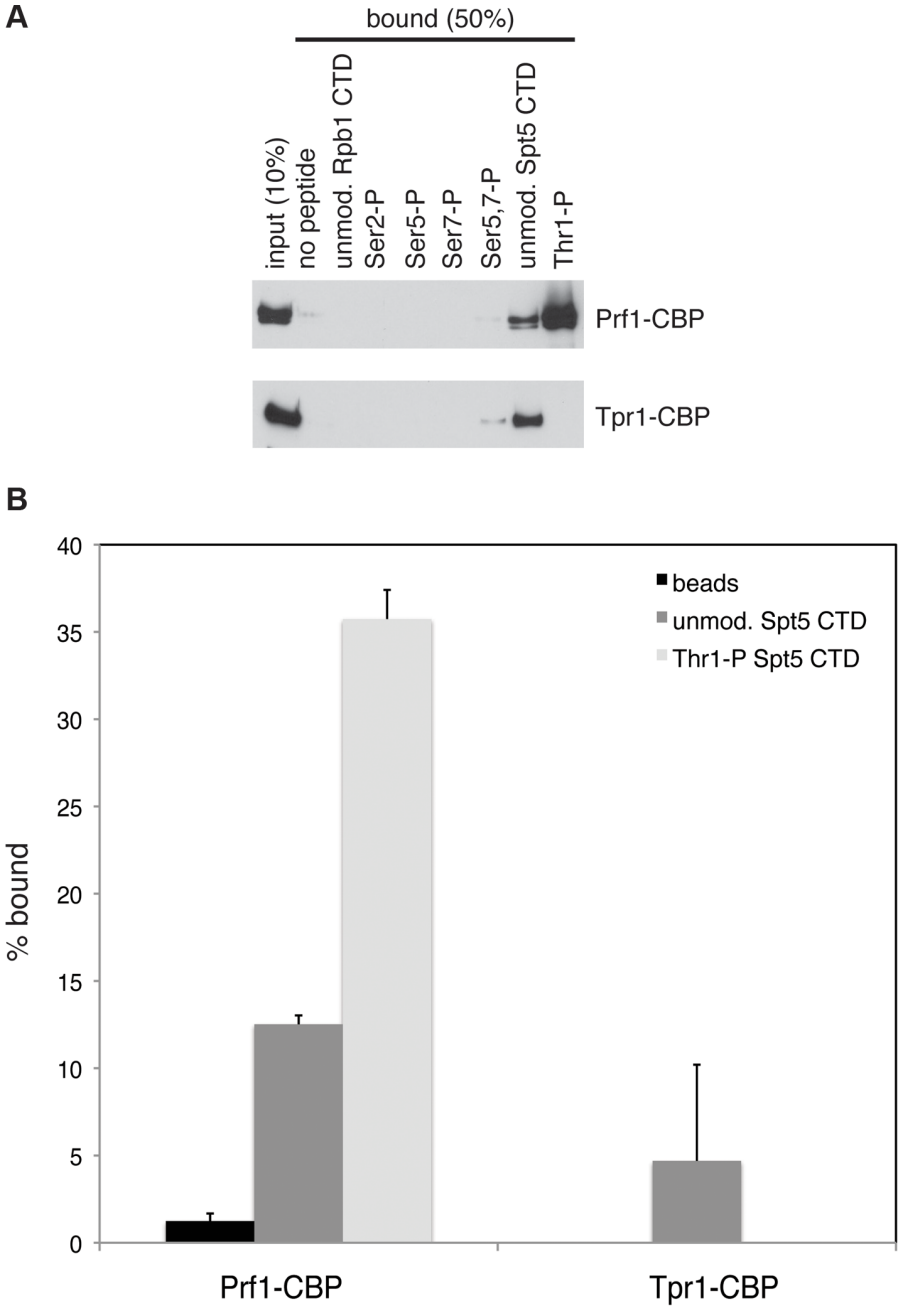
Direct and specific association of Prf1/Rtf1 with the phosphorylated Spt5 CTD. (**A**) The indicated biotinylated peptides were immobilized on streptavidin magnetic beads and incubated with purified Prf1-CBP (top) or Tpr1-CBP (bottom). Input and bound material was analyzed by SDS-PAGE and western blotting with a TAP tag antibody as shown. (**B**) Quantification of binding experiments represented in (A). Error bars denote standard deviations from three independent experiments.

The marked dependence of Prf1 chromatin association on Spt5-P led us to test whether this could reflect a direct interaction between the Spt5 CTD and Prf1. Biotinylated peptides corresponding to the unmodified or the Thr1-P form of the Spt5 CTD were coupled to streptavidin beads and incubated with TAP-purified, native Prf1 or PAF. We detected binding of Prf1 (∼10% bound) to the unmodified Spt5 CTD peptide that was enhanced by phosphorylation (∼35% bound) (Figures 5A and 5B). This interaction was specific, as we detected no binding of Prf1 to Rpb1 CTD phospho-peptides modified at Ser2, Ser5, Ser7, or Ser5 and Ser7. Given that TAP-purified Prf1 is not visibly associated with other proteins (Figure 1), this argues that there is a direct interaction between Prf1 and Spt5-P.

PAF (purified from the *tpr1-TAP* strain) also bound (∼5% of total) to the unmodified Spt5 CTD peptide but failed to bind detectably to the Spt5-P peptide, suggesting that, in vitro, phosphorylation inhibits PAF association with the Spt5 CTD (Figures 5A and 5B). Similar results were obtained with PAF purified from the *paf1-TAP* strain (Figure S7). Thus, we presume that the modest negative effect of the *spt5-T1A* mutation on Tpr1-TAP crosslinking to active genes was due to another Spt5 CTD-binding factor. Consistent with our ChIP data, we did not detect PAF association with unmodified or singly modified Rpb1 CTD peptides, and detected weak association with the peptide that was doubly phosphorylated at Ser5 and Ser7.

### Evidence that PAF and Prf1 have opposing effects on RNAPII elongation

We previously argued that the relationship between Cdk9 and H2Bub1 is mediated by two Cdk9 pathways, one involving Spt5-P and another involving Spt5-P and an additional target [11]. Our data suggested the two pathways were functionally opposed. We wished to test whether the Prf1 and PAF pathways described here might mediate these opposing functions.

**Figure 6:**
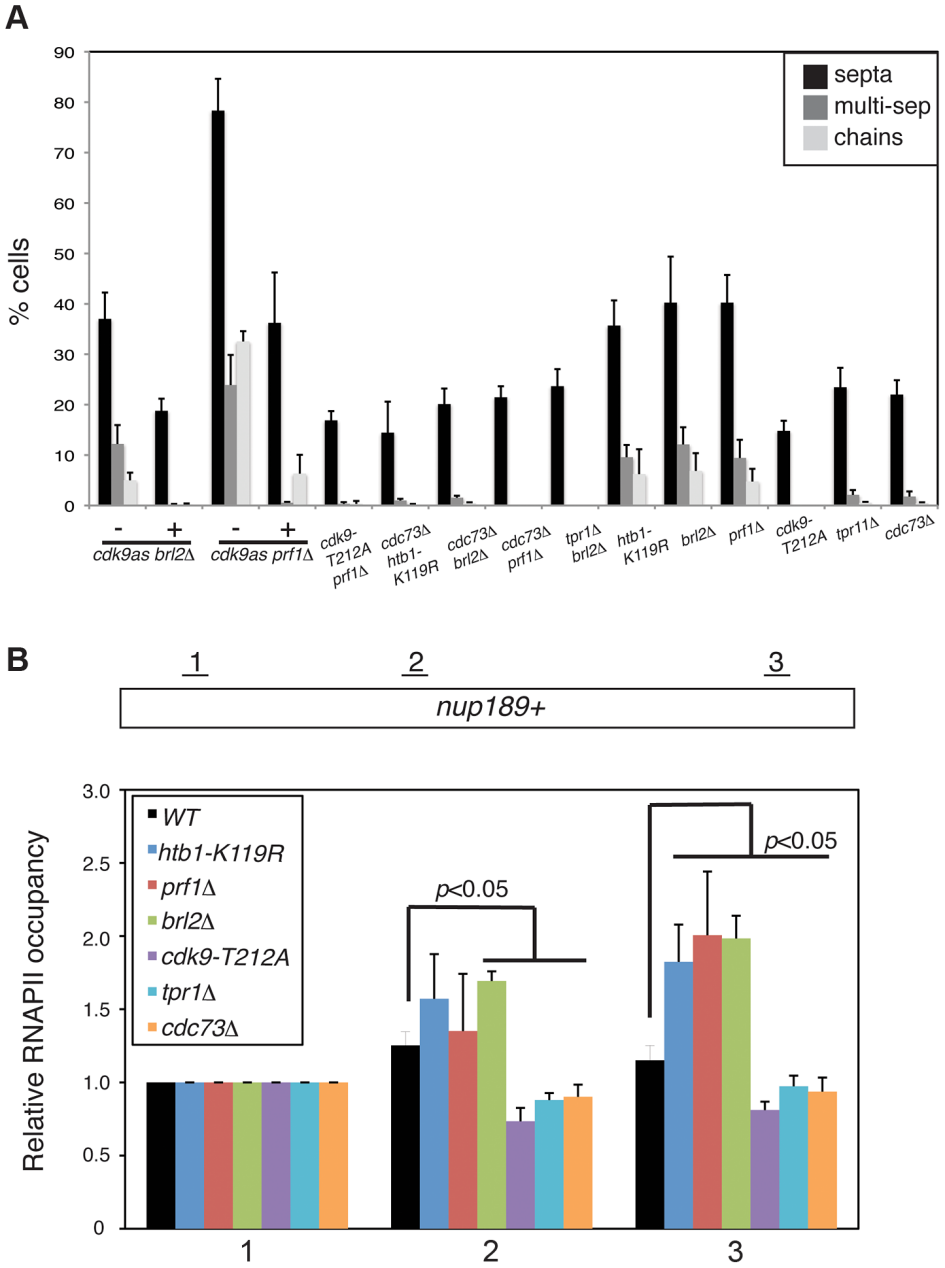
Prf1 and PAF pathways have opposing biological effects. (**A**) Quantification of the abnormal septation patterns in the indicated strains. Strains carrying the *cdk9^as^* allele were cultured in DMSO (−) or 2 µM 3-MB-PP1 (+) for 15 hours prior to fixation for microscopy. Error bars denote standard deviations from 3 independent experiments. (**B**) ChIP of RNAPII was carried out in the indicated strains and quantified by qPCR using primers specific to the *nup189* ^+^ gene. Values were normalized to that for primer pair 1. Error bars denote standard deviations from three independent experiments. Significant differences from wild-type values (unpaired t-test) are indicated.

We first assessed genetic interactions between *prf1*Δ and reduction of Cdk9 activity. We introduced the *cdk9^as^* allele into the *prf1*Δ background and analyzed double mutants by DAPI/calcofluor staining after inhibition of Cdk9^as^ with 3-MB-PP1. Cdk9^as^ inhibition strongly suppressed the cell morphology and septation phenotypes caused by *prf1*Δ (as it did for *brl2*Δ; Figure 6A). A hypomorphic *cdk9-T212A* mutation similarly rescued these phenotypes. These results indicate that the cell morphology and septation defects in *prf1*Δ, *brl2*Δ, and *htb1-K119R* strains depend upon Cdk9 activity, and suggest that Prf1, Brl2, and H2Bub1 itself act to limit some aspect of Cdk9 function.

We next determined whether PAF mutations, which phenocopy *cdk9-T212A*, could also suppress the cell morphology and septation phenotypes of *htb1-K119R*, *brl2*Δ, and *prf1*Δ mutants. We found that *cdc73*Δ, when combined with *htb1-K119R*, *brl2*Δ, or *prf1*Δ, suppressed aberrant septation. Similarly, *brl2*Δ septation phenotypes were effectively suppressed by *tpr1*Δ (Figure 6A). The dependence of these phenotypes on both Cdk9 activity and PAF is consistent with the Prf1 pathway acting in opposition to the PAF pathway.

We also tested whether *htb1-K119R*, *brl2*Δ, and *prf1*Δ could reciprocally suppress *cdk9* and PAF mutant phenotypes. None of these mutations affected the growth of *cdk9* or PAF mutants on minimal media. Whereas *htb1-K119R* suppressed the MPA sensitivity of *cdk9-T212A*, *prf1*Δ did not, and *brl2*Δ enhanced this phenotype (Figure S8). These results indicate that the Prf1 pathway does not generally limit PAF pathway function.

In order to ascertain how these two pathways regulate RNAPII elongation, we carried out RNAPII ChIP experiments in strains harboring mutations in either the Prf1 or PAF pathways. We quantified the ChIP by qPCR with primers in *nup189* ^+^ and *SPBC354.10* ^+^, genes that exemplify the 3′ shift in RNAPII distribution that we documented previously [11]. Consistent with our previous data, the *htb1-K119R* mutation led to an increase in RNAPII occupancy near the 3′ end of the *nup189* ^+^ gene relative to the 5′ end (Figures 6B and S9A). A similar effect was also observed for *brl2*Δ and *prf1*Δ mutations, implying that the 3′ RNAPII shift is characteristic of the Prf1 pathway. In contrast, *cdk9-T212A*, *cdc73*Δ, and *tpr1*Δ mutations led to a relative decrease in RNAPII density toward the 3′ end of the gene, consistent with defective elongation (Figure 6B). The effects on RNAPII distribution were generally less significant at *SPBC354.10* ^+^, but we nonetheless observed a similar pattern in which RNAPII occupancy increased toward the 3′ end of the gene in Prf1 pathway mutants and decreased or was unaffected in PAF pathway mutants (Figure S9A).

**Figure 7:**
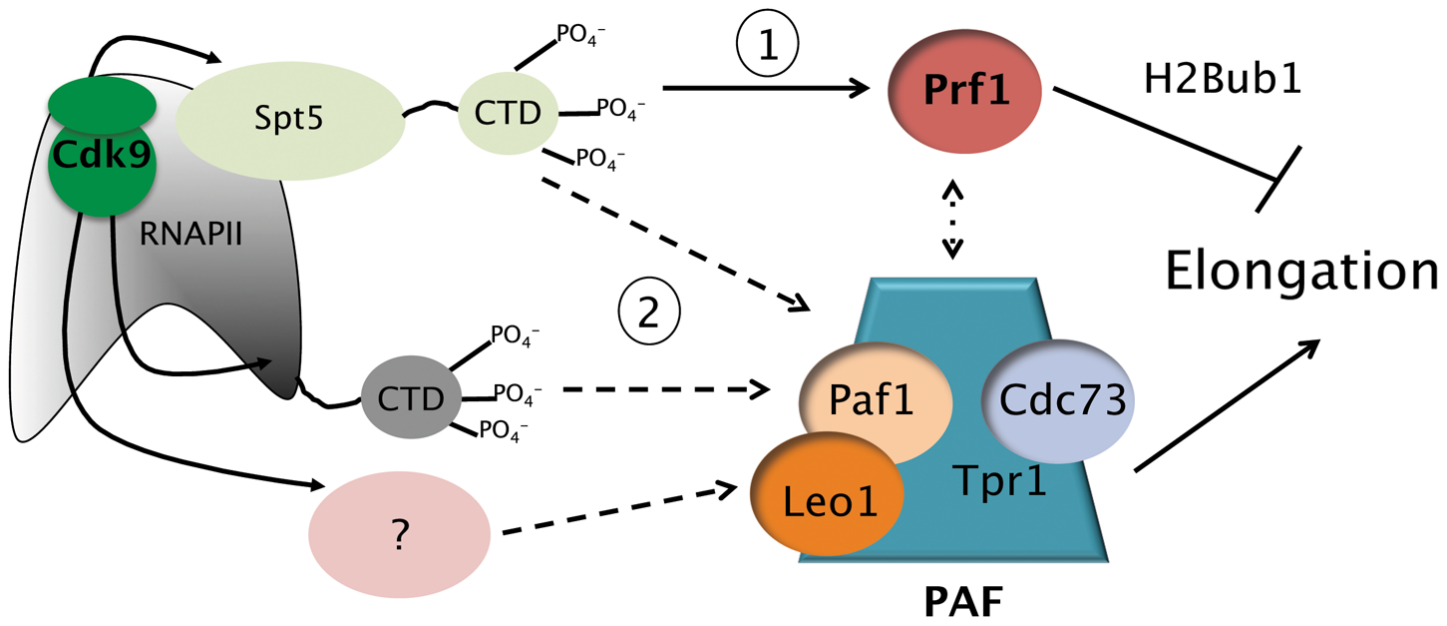
Model depicting the roles of the Prf1/Rtf1 and PAF pathways in RNAPII elongation. The Prf1 pathway, involving direct association of Prf1 with phosphorylated Spt5 CTD, is labeled “1.” The PAF pathway, involving multiple Cdk9 targets, is labeled “2.” Potential crosstalk between the two pathways is indicated by the broken double arrow. See text for details.

We also found that the Prf1 and PAF pathway mutations differentially affected the steady-state levels of mRNA produced from these genes. PAF pathway mutants exhibited modest decreases in mRNA levels, whereas no change or modest increases were observed in the Prf1 pathway mutant strains (Figure S9B). These changes are likely to reflect altered RNAPII elongation, since the absolute levels of RNAPII occupancy near the 5′ ends of these genes did not differ between Prf1 and PAF pathway mutant strains (Figure S9C). Overall, our data suggest that the PAF and Prf1 pathways have differential effects on behavior of the RNAPII elongation complex (Figure 7).

## Discussion

Cdk9 influences multiple aspects of RNAPII transcription. Our results here reveal that phosphorylation of the Spt5 CTD by Cdk9 directly and specifically recruits the conserved transcriptional regulator Prf1/Rtf1 to transcribed chromatin. We further define a second Cdk9 pathway, involving phosphorylation of at least one additional target, which recruits the PAF complex. We show that these two pathways have distinct and opposing functions, suggesting that balanced action of Cdk9 pathways is a feature of RNAPII elongation.

### Prf1/Rtf1 and PAF are biochemically separate in *S. pombe*

Prf1/Rtf1 and PAF do not form a stable complex in *S. pombe* whole-cell extracts, a finding consistent with the properties of these proteins in metazoans. Biochemical studies of human Rtf1 have shown that it can be assembled into a complex with PAF in vitro, and that a C-terminal fragment of Rtf1 can be detected in association with PAF purified from nuclear extracts [22]. Although the physiological significance of these findings has not been explored, they suggest the possibility of a regulated interaction between full-length Rtf1 and PAF. Modulation of this interaction could underlie the shared and distinct functions of Rtf1 and PAF in different biological contexts.

The similarities we have found between the biochemical properties of Prf1 and PAF in fission yeast and those of their metazoan orthologs underscore the utility of the *S. pombe* system as a model for study of these factors. *S. pombe* Prf1 and PAF also show functional resemblance to their human counterparts in that they promote the H2Bub1/H3K4me histone modification axis but not H3K36me, a modification that is PAF-dependent in *S. cerevisiae* [26], [43].

We found that individual subunits within *S. pombe* PAF were functionally distinct: loss of either Tpr1 or Cdc73 was associated with more severe phenotypic consequences in our assays than loss of Paf1 or Leo1. This contrasts with PAF functional organization in *S. cerevisiae*, where *paf1*Δ causes pleiotropic phenotypes [42]. Further investigation into physical interactions between individual PAF subunits and other factors will help to clarify these functional differences.

### Prf1/Rtf1 is a direct downstream effector of phosphorylated Spt5 CTD

Our data show that Prf1/Rtf1 directly and specifically associates with the phosphorylated Spt5 CTD, a finding that is consistent with recent reports on budding yeast and human orthologs of this protein [30], [31], [54]. In vitro, budding yeast Rtf1 was found to bind Spt5-P and the phosphorylated Rpb1 CTD with comparable affinities, and Rtf1 mutations that abrogate its interaction with Spt5-P prevented the association of multiple PAF subunits with transcribed chromatin in vivo [30], [54]. The budding yeast Ctr9 and Cdc73 proteins could also interact with phosphorylated peptides corresponding to either the Spt5 CTD or the Rpb1 CTD [30]. The apparently overlapping mechanisms involved in recruitment of Rtf1 and other PAF components to chromatin in budding yeast are in line with the robust association of Rtf1 with PAF found in this organism. Our findings in *S. pombe* point to a specific and primary role for the Prf1/Spt5-P interaction in Prf1 recruitment to transcribed chromatin, and suggest an alternate recruitment mechanism for PAF.

The direct interaction between Prf1/Rtf1 and Spt5-P helps explain the critical role of Spt5-P in establishment of H2Bub1 levels. This interaction also raises mechanistic questions regarding the reciprocal dependence of Spt5-P on H2Bub1. Loss of Spt5-P in the *htb1-K119R* mutant is accompanied by a reduction in crosslinking of Cdk9 to transcribed chromatin [11]. In the *prf1*Δ strain, it is possible that loss of Spt5-P is also attributable to the absence of the Prf1/Spt5-P interaction, which could provoke dephosphorylation of Spt5-P.

The *spt5-T1A* mutation did not result in cell morphology and septation defects observed in *prf1*Δ, *brl2*Δ, or *htb1-K119R* mutant strains, suggesting that some Prf1 function is maintained in the absence of Spt5 CTD phosphorylation [11]. This is consistent with our ChIP results, which demonstrated a substantial but incomplete loss of Prf1 chromatin recruitment in *spt5-T1A* mutant strains. Since residual Prf1 recruitment was further reduced by Cdk9^as^ inhibition, other Cdk9 targets are likely to play some role in this process, although the bypass of Cdk9 dependence in the *spt5-T1E* mutant suggests that the Spt5 CTD is the primary Cdk9 target involved. Multiple domains of *S. cerevisiae* Rtf1 can be independently recruited to chromatin, indicating that mechanisms other than Spt5 phosphorylation are also important for Rtf1 chromatin association [31], [46], [55]. It is also possible that Prf1 may retain some function even when it is not stably bound to chromatin, as has been observed for the *S. cerevisiae* PAF complex [56].

### PAF association with transcribed chromatin requires phosphorylation of multiple targets by Cdk9

We found that PAF recruitment to chromatin in vivo was only modestly sensitive to Spt5 CTD phosphorylation at both transcribed loci tested. Since we found no interaction between the purified PAF complex and Spt5-P peptide in vitro, we presume that this reflects an indirect effect on PAF association with chromatin mediated by another factor that binds to Spt5-P. This is unlikely to be Prf1, as PAF chromatin association is not affected by the *spt5-T1E* mutation (unlike that of Prf1).

Because crosslinking of Tpr1-TAP and Paf1-TAP to transcribed loci was dramatically reduced by Cdk9^as^ inhibition even in an *spt5-T1A* mutant, PAF recruitment must require at least one other Cdk9 target in addition to Spt5. Given that the Rpb1 CTD is an established target of Cdk9 activity, we were surprised to find that simultaneous elimination of Ser2 and Ser7 in the Rpb1 CTD repeat, or removal of Ser5, had no substantial effect on PAF recruitment in vivo. This could reflect PAF association with forms of the Rpb1 CTD repeat phosphorylated on both Ser5 and another residue, as has been suggested by recent biochemical experiments with budding yeast PAF components [30]. Alternatively, the role of the Rpb1 CTD may be redundant with the Spt5 CTD [19], [57]. Pleiotropic phenotypes associated with mutant combinations that include Rpb1-Ser5 have precluded thorough genetic tests of these possibilities. Recruitment of the PAF complex to chromatin may also involve another, as yet unidentified, Cdk9 target.

### Balanced action of Prf1/Rtf1 and PAF pathways in RNAPII elongation

Septation and cell morphology phenotypes of Prf1 pathway mutants were reduced by *cdk9-T212A*, *cdc73*Δ, and *tpr1*Δ mutations, indicating that these components act aberrantly in the absence of the Prf1 pathway. Prf1 pathway mutations did not generally suppress *cdk9* or PAF mutant phenotypes, suggesting that the Prf1 pathway has a specialized role in limiting certain aspects of PAF pathway function.

ChIP of RNAPII reveals differential effects of the two pathways on RNAPII distribution within the *nup189* ^+^ and *SPBC354.10* ^+^ gene-coding regions, with the PAF pathway promoting RNAPII occupancy at promoter-distal sequences and the Prf1 pathway limiting occupancy at these locations. This observation supports our earlier studies on the opposing effects of Cdk9 activity and H2Bub1 on intragenic RNAPII distribution, and implies that these findings also characterize the two Cdk9 pathways we have described.

We propose that Cdk9-dependent recruitment of PAF, through phosphorylation of the Spt5 CTD and other targets, promotes RNAPII elongation through gene coding regions, as supported by our RNAPII ChIP and mRNA expression data. Spt5 CTD phosphorylation also recruits Prf1/Rtf1 and promotes formation of H2Bub1, which may act as an auto-regulatory mechanism to balance the positive effects of the PAF pathway. Previous studies documenting a positive role for PAF in elongation have shown Rtf1 to be dispensable for this function, consistent with the distinctive behavior of Prf1 in *S. pombe* [21]–[23]. Although we did not observe significant effects of the Prf1 pathway mutations on expression of *nup189^+^* and *SPBC354.10^+^* (with the exception of a slight increase for *SPBC354.10^+^* in the *htb1-K119R* mutant), it is possible that loss of the putative Cdk9 auto-regulatory function in these mutants would be associated with defective mRNA processing or export, rather than a change in mRNA levels per se. Further elucidation of the mechanisms through which these pathways affect gene expression will be an important goal of future research.

A negative role for the Prf1 pathway in elongation could be related to the functions of H2Bub1 in directing nucleosome assembly and histone deacetylation within gene coding regions [58]–[61]. Indeed, H2Bub1 and the Hos2 deacetylase complex have been linked to reduced RNAPII processivity in budding yeast [61].

It is notable that an interaction between PAF and the elongation factor TFIIS that stimulates elongation in mammalian cells is antagonized by the H2Bub1 ubiquitin ligase RNF20 [62]. Although we find no role for TFIIS in opposing H2Bub1 functions in *S. pombe* (data not shown), a role for the Prf1 pathway and H2Bub1 in altering PAF function in elongation may be conserved.

In metazoans, Cdk9 activity has an essential role in release of RNAPII from promoter-proximal pause sites. Phosphorylation of multiple targets, including the Rpb1 CTD, Spt5, and NELF, is thought to drive RNAPII from the pause, implying coordinate action of Cdk9 phosphorylation events in pause release [4], [63]. Interestingly, the experiments defining the role of PAF in P-TEFb-dependent pause release in vitro used PAF complex devoid of Rtf1 [21]. We suggest that the P-TEFb pause release function in metazoans, which involves multiple Cdk9 targets and PAF, is analogous to the PAF pathway we have documented in *S. pombe*. The metazoan analog of the Prf1 pathway might involve Rtf1 recruitment via Spt5-P subsequent to pause release, as part of a mechanism to keep the stimulatory effects of PAF on elongation in check. Thus, the roles of Cdk9 targets in regulating the RNAPII elongation complex may diverge during the course of elongation.

## Materials and Methods

### Strains and media

*S. pombe* strains used in this study are listed in Table S2. Liquid cultures used standard YES media (yeast extract 5 g/L, D-glucose 30 g/L, supplemented with 250 mg/L each of histidine, leucine, adenine and uracil). Minimal media (EMM) was purchased from MP Biomedicals. EMM complete was supplemented with 250 mg/L each of histidine, leucine, adenine, and uracil. Mycophenolic acid (MPA; Bioshop) was dissolved in dimethyl sulfoxide (DMSO) and used at a final concentration of 25 µg/mL. The TAP and 13xmyc tags were introduced at the relevant chromosomal loci as described previously [64]. All other genetic manipulations were carried out using standard methods [65].

### Tandem affinity purification and mass spectrometry

Paf1-TAP, Prf1-TAP and Tpr1-TAP were purified from whole cell extracts as described [48]. For mass spectrometry analysis of polypeptides excised from polyacrylamide gels, purified material was concentrated by addition of 0.02% sodium deoxycholate, incubation on ice for 30 min, and precipitation with trichloroacetic acid (15% final concentration) overnight. The precipitate was collected by centrifugation, washed twice with ice-cold ethanol and then resuspended with 10 µl of SDS-PAGE sample buffer. Proteins were resolved by SDS-PAGE on a 4–20% gradient acrylamide gel and visualized by Coomassie (Bio-rad) or silver staining before mass spectrometry analysis.

To analyze the purified material in solution, the TAP purification procedure was modified as follows: Glycerol and detergent were omitted from the final wash and elution steps, and EGTA was replaced with EDTA in the elution buffer. The eluted material was subjected to mass spectrometry analysis, details of which are provided in the Supplemental Material (see Text S1).

For single-step TAP purification, whole cell extracts were incubated with IgG-sepharose beads (GE Healthcare) and the beads were collected and washed as described [48]. Beads were suspended in SDS sample buffer and bound material was analyzed by SDS-PAGE and western blotting.

### Immunological methods

Immunoblotting was performed as previously described [11]. The following commercial antibodies were used: H2Bub1 (Active Motif #39623), H3K4me1 (Abcam #ab8895), H3K4me2 (Abcam #ab32356), H3K4me3 (Abcam #ab8580), H3K36me3 (Abcam #ab9050), histone H3 (Abcam #ab1791), histone H2B (Millipore #07-371), TAP tag (Fisher Scientific #PICAB1001), Rpb1 (8WG16; Covance #MMS-126R-200), Rpb1-Ser2-P (H5; Covance #MMS-129R), myc (9E10; Covance #MMS-150P). Antibodies against *S. pombe* Spt5 and Spt5-P were described previously [11].

### Phenotypic analyses

DAPI/calcofluor staining was carried out as described previously [11]. For spot tests, 4×10^5^ cells were harvested from exponentially growing cultures, and resuspended in YES media. Ten-fold serial dilutions of the culture were spotted onto the appropriate plates and incubated at 30°C for 3–6 days.

### Chromatin immunoprecipitation

ChIP was carried out as previously described [48] with minor modifications. Briefly, 1.5×10^7^ cells grown in YES media were fixed with 1% formaldehyde for 30 min. Glycine (pH 2.5) was added at a final concentration of 125 mM to stop the crosslinking. Cells were washed twice with cold 1XTBS (20 mM Tris-HCl, pH 7.5, 150 mM NaCl, 1 mM Tween-20) and snap frozen in liquid nitrogen. Cell pellets were resuspended in 0.5 mL of lysis buffer [50 mM HEPES, pH 7.6, 150 mM NaCl, 1 mM EDTA, 1% Triton X-100, 0.1% sodium deoxycholate, 1 mM PMSF, protease inhibitor cocktail (Roche)], 200 µl of sterile glass beads were added, and cells were lysed at 4°C using a mini-beat beater (4×30 s with 1 min rests on ice). The lysates were transferred to 1.5 mL tubes and centrifuged at top speed for 15 min to collect the chromatin. Chromatin pellets were resuspended in 1 mL lysis buffer, sonicated for 20 minutes using a Bioruptor waterbath sonicator (Diagenode) (30 s ON/OFF with 2 min rest on ice after 10 min) and centrifuged at top speed for 5 minutes. The supernatant (700–800 µl) was incubated for 2–3 h with either 50 µl of IgG sepharose beads (GE Healthcare)(for TAP immunoprecipitation) or with 15 µl of protein G Dynabeads (Invitrogen) coupled to 8WG16 antibody (for RNAPII immunoprecipitation) at 4°C with rocking. The beads were washed successively with 0.5 mL of each of the following buffers: lysis buffer +0.1% SDS, lysis buffer +0.1% SDS+500 mM NaCl, LiCl buffer (10 mM Tris-HCl, pH 7.5, 1 mM EDTA, 250 mM LiCl, 0.5% sodium deoxycholate, 0.5% NP-40) and TE (10 mM Tris-HCl, pH 7.5, 1 mM EDTA). Chromatin was eluted with 100 µl of elution buffer (50 mM Tris-HCl, pH 7.5, 10 mM EDTA, 1% SDS) at 65°C for 30 min. Reversal of crosslinks, DNA purification, and quantitative PCR were as described [48]. Primers specific for the *act1^+^*, *adh1^+^*, *nup189^+^*, and *SPBC354.10^+^* genes have been described previously [11], [66].

### Peptide-binding assays

15 µl of streptavidin Dynabeads (Invitrogen) were washed three times with 400 µl of 1xPBS/0.1% Triton-X100. Beads were coupled to 50 µg biotinylated peptide in 380 µl of 1xPBS. Incubation was at room temperature for 3 h with rocking. Spt5 CTD peptides were synthesized at the Keck Foundation Research Laboratory (Yale University) and had the sequence [biotin]-GSKTPAWNSGSKTPAWNS. In the phospho-peptide both Thr residues were phosphorylated. Rpb1 CTD peptides were a generous gift from Francois Robert (IRCM, Montreal) and had the following sequences: [biotin]-YSPTSPSYSPTSPSYSPTSPS, [biotin]-YSPTSPSY(pSer)PTSPSYSPTSPS, [biotin]-SPTSPSYSPT(pSer)PSYSPTSPSY, [biotin]-TSPSYSPTSP(pSer)YSPTSPSYSP, [biotin]-PTSPSYSPT(pSer)P(pSer)YSPTSPSYS. Beads were washed twice with washing buffer (20 mM HEPES pH 7.6, 20% v/v glycerol, 1 mM EDTA, 0.1% Triton X-100, 250 mM KOAc, 10 mM β-glycero-3-phosphate, 1 mM PMSF) and resuspended with 150 µl of the binding buffer (20 mM HEPES pH 7.6, 0.1% Triton X-100, 1 mM PMSF, 10 mM β-glycero-3-phosphate). Binding reactions contained 20 mM Hepes pH 7.6, 0.075% Triton X-100, 0.75 mM PMSF, 7.5 mM β-glycero-3-phosphate, 5% glycerol, 37.5 mM KOAc, 2.5 mM MgOAc, 0.25 mM EDTA, 0.25 mM DTT and 50–200 ng of purified factors (∼50 ng for Prf1-TAP or 200 ng for Paf1-TAP and Tpr1-TAP) in a volume of 200 µl. The mixtures were incubated at 4°C for 1 h with shaking. Beads were washed 4 times with 1 mL of washing buffer, resuspended with 20 µl of SDS-PAGE sample buffer, boiled for 5 minutes at 95°C, and subjected to SDS-PAGE and immunoblotting with a TAP antibody. Signal intensities were quantified using ImageJ software and used to calculate percent bound.

## Supporting Information

Figure S1 TAP-tagged Paf1, Tpr1, and Prf1 proteins are functional. Whole-cell extracts from the indicated TAP-tagged strains were analyzed by SDS-PAGE and western blotting with the indicated antibodies.
(TIF)

Figure S2 PAF and Prf1 have shared functions in promoting co-transcriptional methylation of histone H3 lysine 4. Whole-cell extracts from strains of the indicated genotypes were analyzed by SDS-PAGE and western blotting with the indicated antibodies.
(TIF)

Figure S3 Inhibition of Cdk9 does not affect total levels of PAF or Prf1 and does not dramatically alter RNAPII occupancy at the constitutive *act1* ^+^ and *adh1* ^+^ genes. (**A**) The indicated TAP-tagged strains were treated with DMSO (−) or 20 µM 3-MB-PP1 (+) for 2 hours prior to whole-cell extract preparation and analysis by SDS-PAGE and western blotting. Levels of TAP-tagged proteins were assessed with the TAP antibody. (**B**) ChIP of RNAPII was carried out in the *cdk9^as^* strain and quantified by qPCR using primers specific to the *nup189* ^+^ gene. Error bars denote standard deviations from three independent experiments.
(TIF)

Figure S4 Prf1 and PAF are recruited to chromatin via alternate mechanisms. (**A**) **and** (**B**). Strains used for ChIP with IgG resin (recognizing the TAP tag) are indicated at the bottom. All strains also harbored the *cdk9^as^* allele and were treated with either DMSO (−) or 20 µM 3-MB-PP1 (+) for two hours before crosslinking for ChIP. Assays were quantified by qPCR using primers specific for the *adh1* ^+^ gene. Length of the gene coding region (in base pairs) and positions of PCR amplicons are as indicated at the top. Error bars denote standard deviations from 2–3 independent experiments.
(TIF)

Figure S5 Prf1 and PAF differentially regulate phosphorylation of Spt5 CTD and Rpb1-Ser2. (**A**) Whole-cell extracts from strains of the indicated genotypes were analyzed by SDS-PAGE and western blotting with either the 8WG16 antibody (to detect total RNAPII) or the H5 antibody (to detect RNAPII phosphorylated at Rbp1-Ser2). Relative band intensities were quantified using ImageJ software and the ratios of Spt5-P signal to total Spt5 signal for each strain are plotted. Error bars denote standard deviation from 3 independent experiments and asterisks denote significant differences from the wild-type intensity (*p*<0.05; unpaired t-test). (**B**) As in (A).
(TIF)

Figure S6 Rpb1-Ser5 is not required for recruitment of Prf1 or PAF to chromatin. ChIP was performed on the indicated strains with IgG resin (recognizing the TAP tag). Assays were quantified by qPCR using primers specific for the *act1* ^+^ (left) or *adh1* ^+^ (right) genes. Lengths of the gene coding regions (in base pairs) and positions of PCR amplicons are indicated at the top. Error bars denote standard deviations from 2–3 independent experiments.
(TIF)

Figure S7 Purified Prf1 and PAF complex interact differently with the Spt5 CTD. Material purified from the TAP-tagged strains indicated at the top was incubated with magnetic steptavidin beads alone (“no peptide”) or beads coupled to the indicated biotinylated peptides. Input and bound fractions (50%) were analyzed by SDS-PAGE and western blotting with the TAP tag antibody. Molecular weight markers (in kD) are shown on the left.
(TIF)

Figure S8 Phenotypes of *cdk9* and PAF mutants do not require Prf1, Brl2, or H2Bub1. Five-fold serial dilutions of the indicated strains were spotted on agar plates containing rich media (YES), minimal media (EMM), rich media with dimethyl sulfoxide (YES+DMSO), or rich media with mycophenolic acid (YES+MPA).
(TIF)

Figure S9 Differential effects of Prf1 and PAF pathway mutations on intragenic RNAPII distribution and gene expression. (**A**) ChIP of RNAPII was carried out in the indicated strains and quantified by qPCR using primers specific to the *SPBC354.10* ^+^ gene. Values were normalized to that for primer pair 1. Error bars denote standard deviations from three independent experiments. Significant differences from wild-type values (unpaired t-test) are indicated. (**B**) Levels of mRNA from the indicated genes were quantified by qRT-PCR using primer pair 3 in either *nup189* ^+^ or *SPBC354.10* ^+^ and normalized to the signal for primer pair 3 in *act1* ^+^. The resulting value in the wild-type strain was set to 1. Error bars denote standard deviations from two independent experiments. Significant differences from wild-type values (p<0.05; unpaired t-test) are indicated by asterisks. (**C**) ChIP of RNAPII was carried out in the indicated strains and quantified by qPCR using primer pair 1 in either *nup189* ^+^ or *SPBC354.10* ^+^. Values were normalized to that for primer pair 3 in *act1* ^+^. Error bars denote standard deviations from three independent experiments. Significant differences from wild-type values (p<0.05; unpaired t-test) are indicated by asterisks.
(TIF)

Table S1 Mass spectrometry analysis of Tpr1-TAP and Prf1-TAP purifications. Shown are all proteins identified with a confidence level of 95% or greater in at least one of the samples analyzed. PAF complex components and Prf1 are highlighted in yellow.
(PDF)

Table S2 *S. pombe* strains used in this study.
(PDF)

Text S1 Supplemental materials and methods.
(PDF)

## References

[1] Zhou Q, Li T, Price DH (2012) RNA polymerase II elongation control. Annu Rev Biochem 81: 119–143. 22404626 10.1146/annurev-biochem-052610-095910 PMC4273853

[2] Marshall NF, Price DH (1995) Purification of P-TEFb, a transcription factor required for the transition into productive elongation. J Biol Chem 270: 12335–12338. 7759473 10.1074/jbc.270.21.12335

[3] Wada T, Takagi T, Yamaguchi Y, Watanabe D, Handa H (1998) Evidence that P-TEFb alleviates the negative effect of DSIF on RNA polymerase II-dependent transcription in vitro. Embo J 17: 7395–7403. 9857195 10.1093/emboj/17.24.7395 PMC1171084

[4] Yamada T, Yamaguchi Y, Inukai N, Okamoto S, Mura T, et al (2006) P-TEFb-mediated phosphorylation of hSpt5 C-terminal repeats is critical for processive transcription elongation. Mol Cell 21: 227–237. 16427012 10.1016/j.molcel.2005.11.024

[5] Yamaguchi Y, Takagi T, Wada T, Yano K, Furuya A, et al (1999) NELF, a multisubunit complex containing RD, cooperates with DSIF to repress RNA polymerase II elongation. Cell 97: 41–51. 10199401 10.1016/s0092-8674(00)80713-8

[6] Murray S, Udupa R, Yao S, Hartzog G, Prelich G (2001) Phosphorylation of the RNA polymerase II carboxy-terminal domain by the Bur1 cyclin-dependent kinase. Mol Cell Biol 21: 4089–4096. 11390638 10.1128/MCB.21.13.4089-4096.2001 PMC87070

[7] Pei Y, Du H, Singer J, St Amour C, Granitto S, et al (2006) Cyclin-dependent kinase 9 (Cdk9) of fission yeast is activated by the CDK-activating kinase Csk1, overlaps functionally with the TFIIH-associated kinase Mcs6, and associates with the mRNA cap methyltransferase Pcm1 in vivo. Mol Cell Biol 26: 777–788. 16428435 10.1128/MCB.26.3.777-788.2006 PMC1347026

[8] Pei Y, Schwer B, Shuman S (2003) Interactions between fission yeast Cdk9, its cyclin partner Pch1, and mRNA capping enzyme Pct1 suggest an elongation checkpoint for mRNA quality control. J Biol Chem 278: 7180–7188. 12475973 10.1074/jbc.M211713200

[9] Pei Y, Shuman S (2003) Characterization of the Schizosaccharomyces pombe Cdk9/Pch1 protein kinase: Spt5 phosphorylation, autophosphorylation, and mutational analysis. J Biol Chem 278: 43346–43356. 12904290 10.1074/jbc.M307319200

[10] Keogh MC, Podolny V, Buratowski S (2003) Bur1 kinase is required for efficient transcription elongation by RNA polymerase II. Mol Cell Biol 23: 7005–7018. 12972617 10.1128/MCB.23.19.7005-7018.2003 PMC193923

[11] Sanso M, Lee KM, Viladevall L, Jacques PE, Page V, et al (2012) A Positive Feedback Loop Links Opposing Functions of P-TEFb/Cdk9 and Histone H2B Ubiquitylation to Regulate Transcript Elongation in Fission Yeast. PLoS Genet 8: e1002822. 22876190 10.1371/journal.pgen.1002822 PMC3410854

[12] Viladevall L, St Amour CV, Rosebrock A, Schneider S, Zhang C, et al (2009) TFIIH and P-TEFb coordinate transcription with capping enzyme recruitment at specific genes in fission yeast. Mol Cell 33: 738–751. 19328067 10.1016/j.molcel.2009.01.029 PMC2693121

[13] Peterlin BM, Price DH (2006) Controlling the elongation phase of transcription with P-TEFb. Mol Cell 23: 297–305. 16885020 10.1016/j.molcel.2006.06.014

[14] Hsin JP, Manley JL (2012) The RNA polymerase II CTD coordinates transcription and RNA processing. Genes Dev 26: 2119–2137. 23028141 10.1101/gad.200303.112 PMC3465734

[15] Zhou K, Kuo WH, Fillingham J, Greenblatt JF (2009) Control of transcriptional elongation and cotranscriptional histone modification by the yeast BUR kinase substrate Spt5. Proc Natl Acad Sci U S A 106: 6956–6961. 19365074 10.1073/pnas.0806302106 PMC2678430

[16] Liu Y, Warfield L, Zhang C, Luo J, Allen J, et al (2009) Phosphorylation of the transcription elongation factor Spt5 by yeast Bur1 kinase stimulates recruitment of the PAF complex. Mol Cell Biol 29: 4852–4863. 19581288 10.1128/MCB.00609-09 PMC2725703

[17] Shchebet A, Karpiuk O, Kremmer E, Eick D, Johnsen SA (2012) Phosphorylation by cyclin-dependent kinase-9 controls ubiquitin-conjugating enzyme-2A function. Cell Cycle 11: 2122–2127. 22592529 10.4161/cc.20548

[18] Wood A, Schneider J, Dover J, Johnston M, Shilatifard A (2005) The Bur1/Bur2 complex is required for histone H2B monoubiquitination by Rad6/Bre1 and histone methylation by COMPASS. Mol Cell 20: 589–599. 16307922 10.1016/j.molcel.2005.09.010

[19] Schneider S, Pei Y, Shuman S, Schwer B (2010) Separable functions of the fission yeast Spt5 carboxyl-terminal domain (CTD) in capping enzyme binding and transcription elongation overlap with those of the RNA polymerase II CTD. Mol Cell Biol 30: 2353–2364. 20231361 10.1128/MCB.00116-10 PMC2863715

[20] Tomson BN, Arndt KM (2013) The many roles of the conserved eukaryotic Paf1 complex in regulating transcription, histone modifications, and disease states. Biochim Biophys Acta 1829: 116–126. 22982193 10.1016/j.bbagrm.2012.08.011 PMC3541448

[21] Chen Y, Yamaguchi Y, Tsugeno Y, Yamamoto J, Yamada T, et al (2009) DSIF, the Paf1 complex, and Tat-SF1 have nonredundant, cooperative roles in RNA polymerase II elongation. Genes Dev 23: 2765–2777. 19952111 10.1101/gad.1834709 PMC2788331

[22] Kim J, Guermah M, Roeder RG (2010) The human PAF1 complex acts in chromatin transcription elongation both independently and cooperatively with SII/TFIIS. Cell 140: 491–503. 20178742 10.1016/j.cell.2009.12.050 PMC2853908

[23] Rondon AG, Gallardo M, Garcia-Rubio M, Aguilera A (2004) Molecular evidence indicating that the yeast PAF complex is required for transcription elongation. EMBO Rep 5: 47–53. 14710186 10.1038/sj.embor.7400045 PMC1298956

[24] Pavri R, Zhu B, Li G, Trojer P, Mandal S, et al (2006) Histone H2B monoubiquitination functions cooperatively with FACT to regulate elongation by RNA polymerase II. Cell 125: 703–717. 16713563 10.1016/j.cell.2006.04.029

[25] Krogan NJ, Dover J, Wood A, Schneider J, Heidt J, et al (2003) The Paf1 complex is required for histone H3 methylation by COMPASS and Dot1p: linking transcriptional elongation to histone methylation. Mol Cell 11: 721–729. 12667454 10.1016/s1097-2765(03)00091-1

[26] Zhu B, Mandal SS, Pham AD, Zheng Y, Erdjument-Bromage H, et al (2005) The human PAF complex coordinates transcription with events downstream of RNA synthesis. Genes Dev 19: 1668–1673. 16024656 10.1101/gad.1292105 PMC1176003

[27] Penheiter KL, Washburn TM, Porter SE, Hoffman MG, Jaehning JA (2005) A posttranscriptional role for the yeast Paf1-RNA polymerase II complex is revealed by identification of primary targets. Mol Cell 20: 213–223. 16246724 10.1016/j.molcel.2005.08.023

[28] Rozenblatt-Rosen O, Nagaike T, Francis JM, Kaneko S, Glatt KA, et al (2009) The tumor suppressor Cdc73 functionally associates with CPSF and CstF 3′ mRNA processing factors. Proc Natl Acad Sci U S A 106: 755–760. 19136632 10.1073/pnas.0812023106 PMC2615665

[29] Laribee RN, Krogan NJ, Xiao T, Shibata Y, Hughes TR, et al (2005) BUR kinase selectively regulates H3 K4 trimethylation and H2B ubiquitylation through recruitment of the PAF elongation complex. Curr Biol 15: 1487–1493. 16040246 10.1016/j.cub.2005.07.028

[30] Qiu H, Hu C, Gaur NA, Hinnebusch AG (2012) Pol II CTD kinases Bur1 and Kin28 promote Spt5 CTR-independent recruitment of Paf1 complex. Embo J 31: 3494–3505. 22796944 10.1038/emboj.2012.188 PMC3419927

[31] Mayekar MK, Gardner RG, Arndt KM (2013) The recruitment of the Saccharomyces cerevisiae Paf1 complex to active genes requires a domain of Rtf1 that directly interacts with the Spt4-Spt5 complex. Mol Cell Biol 33: 3259–3273. 23775116 10.1128/MCB.00270-13 PMC3753903

[32] Nordick K, Hoffman MG, Betz JL, Jaehning JA (2008) Direct interactions between the Paf1 complex and a cleavage and polyadenylation factor are revealed by dissociation of Paf1 from RNA polymerase II. Eukaryot Cell 7: 1158–1167. 18469135 10.1128/EC.00434-07 PMC2446681

[33] Adelman K, Wei W, Ardehali MB, Werner J, Zhu B, et al (2006) Drosophila Paf1 modulates chromatin structure at actively transcribed genes. Mol Cell Biol 26: 250–260. 16354696 10.1128/MCB.26.1.250-260.2006 PMC1317635

[34] Langenbacher AD, Nguyen CT, Cavanaugh AM, Huang J, Lu F, et al (2011) The PAF1 complex differentially regulates cardiomyocyte specification. Dev Biol 353: 19–28. 21338598 10.1016/j.ydbio.2011.02.011 PMC3075326

[35] Rozenblatt-Rosen O, Hughes CM, Nannepaga SJ, Shanmugam KS, Copeland TD, et al (2005) The parafibromin tumor suppressor protein is part of a human Paf1 complex. Mol Cell Biol 25: 612–620. 15632063 10.1128/MCB.25.2.612-620.2005 PMC543415

[36] Krogan NJ, Kim M, Ahn SH, Zhong G, Kobor MS, et al (2002) RNA polymerase II elongation factors of Saccharomyces cerevisiae: a targeted proteomics approach. Mol Cell Biol 22: 6979–6992. 12242279 10.1128/MCB.22.20.6979-6992.2002 PMC139818

[37] Mueller CL, Jaehning JA (2002) Ctr9, Rtf1, and Leo1 are components of the Paf1/RNA polymerase II complex. Mol Cell Biol 22: 1971–1980. 11884586 10.1128/MCB.22.7.1971-1980.2002 PMC133696

[38] Akanuma T, Koshida S, Kawamura A, Kishimoto Y, Takada S (2007) Paf1 complex homologues are required for Notch-regulated transcription during somite segmentation. EMBO Rep 8: 858–863. 17721442 10.1038/sj.embor.7401045 PMC1973952

[39] Bai X, Kim J, Yang Z, Jurynec MJ, Akie TE, et al (2010) TIF1gamma controls erythroid cell fate by regulating transcription elongation. Cell 142: 133–143. 20603019 10.1016/j.cell.2010.05.028 PMC3072682

[40] He Y, Doyle MR, Amasino RM (2004) PAF1-complex-mediated histone methylation of FLOWERING LOCUS C chromatin is required for the vernalization-responsive, winter-annual habit in Arabidopsis. Genes Dev 18: 2774–2784. 15520273 10.1101/gad.1244504 PMC528897

[41] Ding L, Paszkowski-Rogacz M, Nitzsche A, Slabicki MM, Heninger AK, et al (2009) A genome-scale RNAi screen for Oct4 modulators defines a role of the Paf1 complex for embryonic stem cell identity. Cell Stem Cell 4: 403–415. 19345177 10.1016/j.stem.2009.03.009

[42] Betz JL, Chang M, Washburn TM, Porter SE, Mueller CL, et al (2002) Phenotypic analysis of Paf1/RNA polymerase II complex mutations reveals connections to cell cycle regulation, protein synthesis, and lipid and nucleic acid metabolism. Mol Genet Genomics 268: 272–285. 12395202 10.1007/s00438-002-0752-8

[43] Chu Y, Simic R, Warner MH, Arndt KM, Prelich G (2007) Regulation of histone modification and cryptic transcription by the Bur1 and Paf1 complexes. Embo J 26: 4646–4656. 17948059 10.1038/sj.emboj.7601887 PMC2080810

[44] Keogh MC, Kurdistani SK, Morris SA, Ahn SH, Podolny V, et al (2005) Cotranscriptional set2 methylation of histone H3 lysine 36 recruits a repressive Rpd3 complex. Cell 123: 593–605. 16286008 10.1016/j.cell.2005.10.025

[45] Lenstra TL, Benschop JJ, Kim T, Schulze JM, Brabers NA, et al (2011) The specificity and topology of chromatin interaction pathways in yeast. Mol Cell 42: 536–549. 21596317 10.1016/j.molcel.2011.03.026 PMC4435841

[46] Piro AS, Mayekar MK, Warner MH, Davis CP, Arndt KM (2012) Small region of Rtf1 protein can substitute for complete Paf1 complex in facilitating global histone H2B ubiquitylation in yeast. Proc Natl Acad Sci U S A 109: 10837–10842. 22699496 10.1073/pnas.1116994109 PMC3390850

[47] Eydmann T, Sommariva E, Inagawa T, Mian S, Klar AJ, et al (2008) Rtf1-mediated eukaryotic site-specific replication termination. Genetics 180: 27–39. 18723894 10.1534/genetics.108.089243 PMC2535681

[48] Racine A, Page V, Nagy S, Grabowski D, Tanny JC (2012) Histone H2B ubiquitylation promotes activity of the intact Set1 histone methyltransferase complex in fission yeast. J Biol Chem 287: 19040–19047. 22505722 10.1074/jbc.M112.356253 PMC3365937

[49] Tanny JC, Erdjument-Bromage H, Tempst P, Allis CD (2007) Ubiquitylation of histone H2B controls RNA polymerase II transcription elongation independently of histone H3 methylation. Genes Dev 21: 835–847. 17374714 10.1101/gad.1516207 PMC1838534

[50] Chandrasekharan MB, Huang F, Sun ZW (2010) Histone H2B ubiquitination and beyond: Regulation of nucleosome stability, chromatin dynamics and the trans-histone H3 methylation. Epigenetics 5: 460–468. 20523115 10.4161/epi.5.6.12314 PMC3230548

[51] St Amour CV, Sanso M, Bosken CA, Lee KM, Larochelle S, et al (2012) Separate domains of fission yeast Cdk9 (P-TEFb) are required for capping enzyme recruitment and primed (Ser7-phosphorylated) CTD substrate recognition. Mol Cell Biol 32: 2372–2383. 22508988 10.1128/MCB.06657-11 PMC3434489

[52] Phatnani HP, Jones JC, Greenleaf AL (2004) Expanding the functional repertoire of CTD kinase I and RNA polymerase II: novel phosphoCTD-associating proteins in the yeast proteome. Biochemistry 43: 15702–15719. 15595826 10.1021/bi048364h PMC2879061

[53] Schwer B, Shuman S (2011) Deciphering the RNA polymerase II CTD code in fission yeast. Mol Cell 43: 311–318. 21684186 10.1016/j.molcel.2011.05.024 PMC3142328

[54] Wier AD, Mayekar MK, Heroux A, Arndt KM, Vandemark AP (2013) Structural basis for Spt5-mediated recruitment of the Paf1 complex to chromatin. Proc Natl Acad Sci U S A 110: 17290–17295. 24101474 10.1073/pnas.1314754110 PMC3808610

[55] Warner MH, Roinick KL, Arndt KM (2007) Rtf1 is a multifunctional component of the Paf1 complex that regulates gene expression by directing cotranscriptional histone modification. Mol Cell Biol 27: 6103–6115. 17576814 10.1128/MCB.00772-07 PMC1952162

[56] Mueller CL, Porter SE, Hoffman MG, Jaehning JA (2004) The Paf1 complex has functions independent of actively transcribing RNA polymerase II. Mol Cell 14: 447–456. 15149594 10.1016/s1097-2765(04)00257-6

[57] Lindstrom DL, Hartzog GA (2001) Genetic interactions of Spt4-Spt5 and TFIIS with the RNA polymerase II CTD and CTD modifying enzymes in Saccharomyces cerevisiae. Genetics 159: 487–497. 11606527 10.1093/genetics/159.2.487 PMC1461841

[58] Fleming AB, Kao CF, Hillyer C, Pikaart M, Osley MA (2008) H2B ubiquitylation plays a role in nucleosome dynamics during transcription elongation. Mol Cell 31: 57–66. 18614047 10.1016/j.molcel.2008.04.025

[59] Chandrasekharan MB, Huang F, Sun ZW (2009) Ubiquitination of histone H2B regulates chromatin dynamics by enhancing nucleosome stability. Proc Natl Acad Sci U S A 106: 16686–16691. 19805358 10.1073/pnas.0907862106 PMC2757834

[60] Batta K, Zhang Z, Yen K, Goffman DB, Pugh BF (2011) Genome-wide function of H2B ubiquitylation in promoter and genic regions. Genes Dev 25: 2254–2265. 22056671 10.1101/gad.177238.111 PMC3219230

[61] Weinberger L, Voichek Y, Tirosh I, Hornung G, Amit I, et al (2012) Expression Noise and Acetylation Profiles Distinguish HDAC Functions. Mol Cell 47: 193–202. 22683268 10.1016/j.molcel.2012.05.008 PMC3408861

[62] Shema E, Kim J, Roeder RG, Oren M (2011) RNF20 Inhibits TFIIS-Facilitated Transcriptional Elongation to Suppress Pro-oncogenic Gene Expression. Mol Cell 42: 477–488. 21596312 10.1016/j.molcel.2011.03.011 PMC3099049

[63] Fujinaga K, Irwin D, Huang Y, Taube R, Kurosu T, et al (2004) Dynamics of human immunodeficiency virus transcription: P-TEFb phosphorylates RD and dissociates negative effectors from the transactivation response element. Mol Cell Biol 24: 787–795. 14701750 10.1128/MCB.24.2.787-795.2004 PMC343783

[64] Tasto JJ, Carnahan RH, McDonald WH, Gould KL (2001) Vectors and gene targeting modules for tandem affinity purification in Schizosaccharomyces pombe. Yeast 18: 657–662. 11329175 10.1002/yea.713

[65] Moreno S, Klar A, Nurse P (1991) Molecular genetic analysis of fission yeast Schizosaccharomyces pombe. Methods Enzymol 194: 795–823. 2005825 10.1016/0076-6879(91)94059-l

[66] Guiguen A, Soutourina J, Dewez M, Tafforeau L, Dieu M, et al (2007) Recruitment of P-TEFb (Cdk9-Pch1) to chromatin by the cap-methyl transferase Pcm1 in fission yeast. EMBO J 26: 1552–1559. 17332744 10.1038/sj.emboj.7601627 PMC1829387

