# Supplementary figures and images for: The PAF Complex and Prf1/Rtf1 Delineate Distinct Cdk9-Dependent Pathways Regulating Transcription Elongation in Fission Yeast
Source: PLoS Genet. 2013 Dec 26;9(12):e1004029. doi: 10.1371/journal.pgen.1004029 (PMC3873232; doi:10.1371/journal.pgen.1004029)

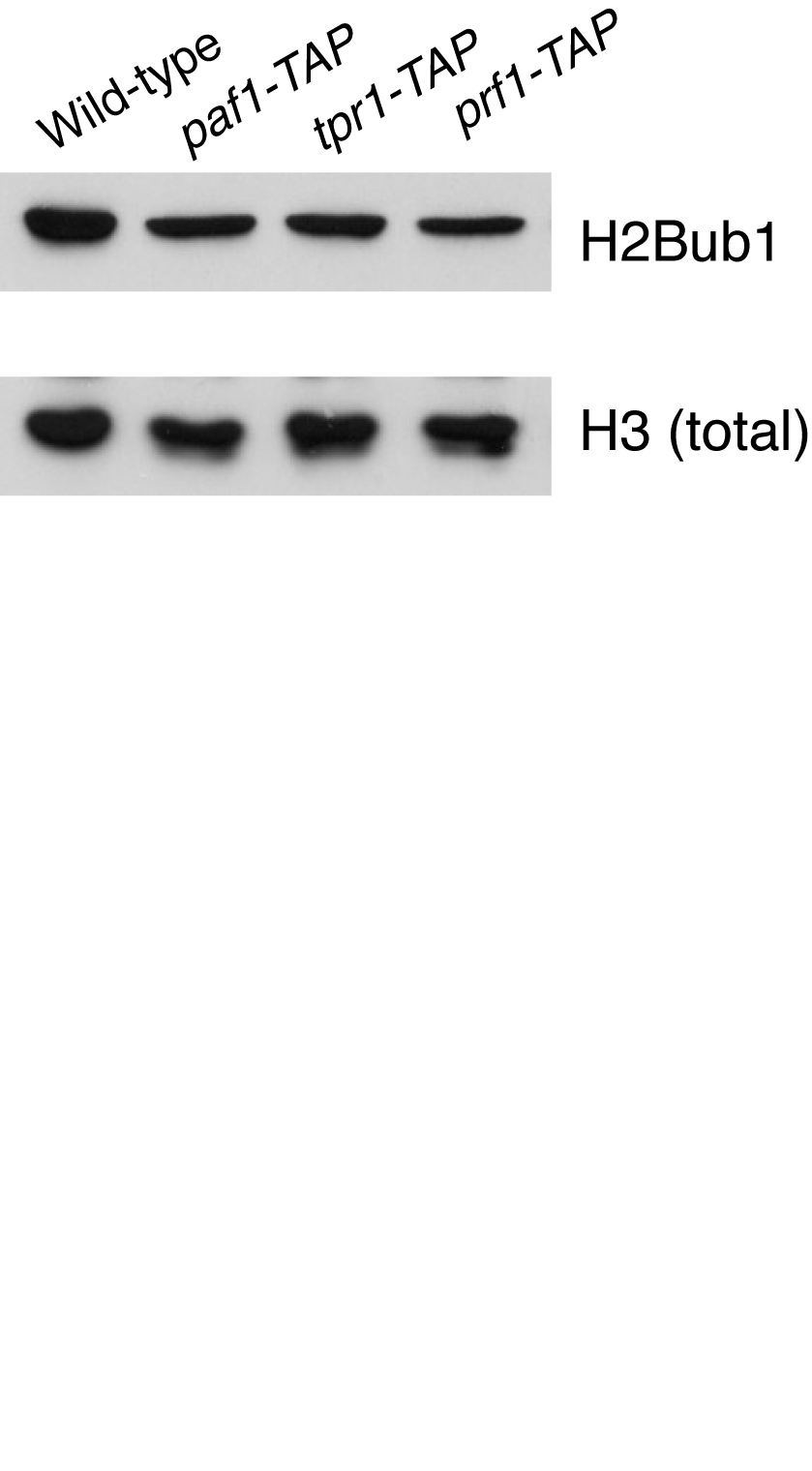

Supplement: Figure S1 — TAP-tagged Paf1, Tpr1, and Prf1 proteins are functional. Whole-cell extracts from the indicated TAP-tagged strains were analyzed by SDS-PAGE and western blotting with the indicated antibodies. (TIF) [file pgen.1004029.s001.tif]

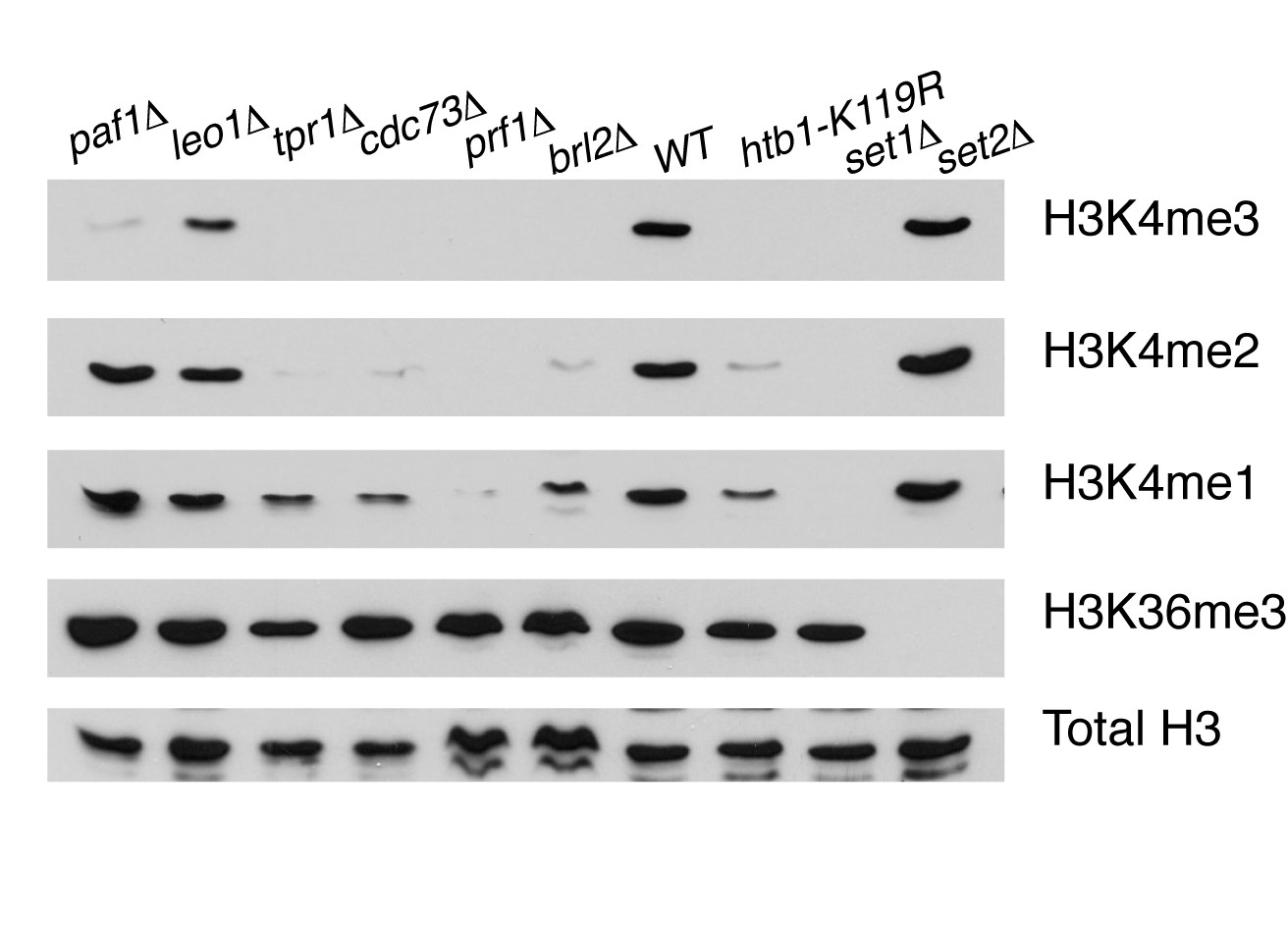

Supplement: Figure S2 — PAF and Prf1 have shared functions in promoting co-transcriptional methylation of histone H3 lysine 4. Whole-cell extracts from strains of the indicated genotypes were analyzed by SDS-PAGE and western blotting with the indicated antibodies. (TIF) [file pgen.1004029.s002.tif]

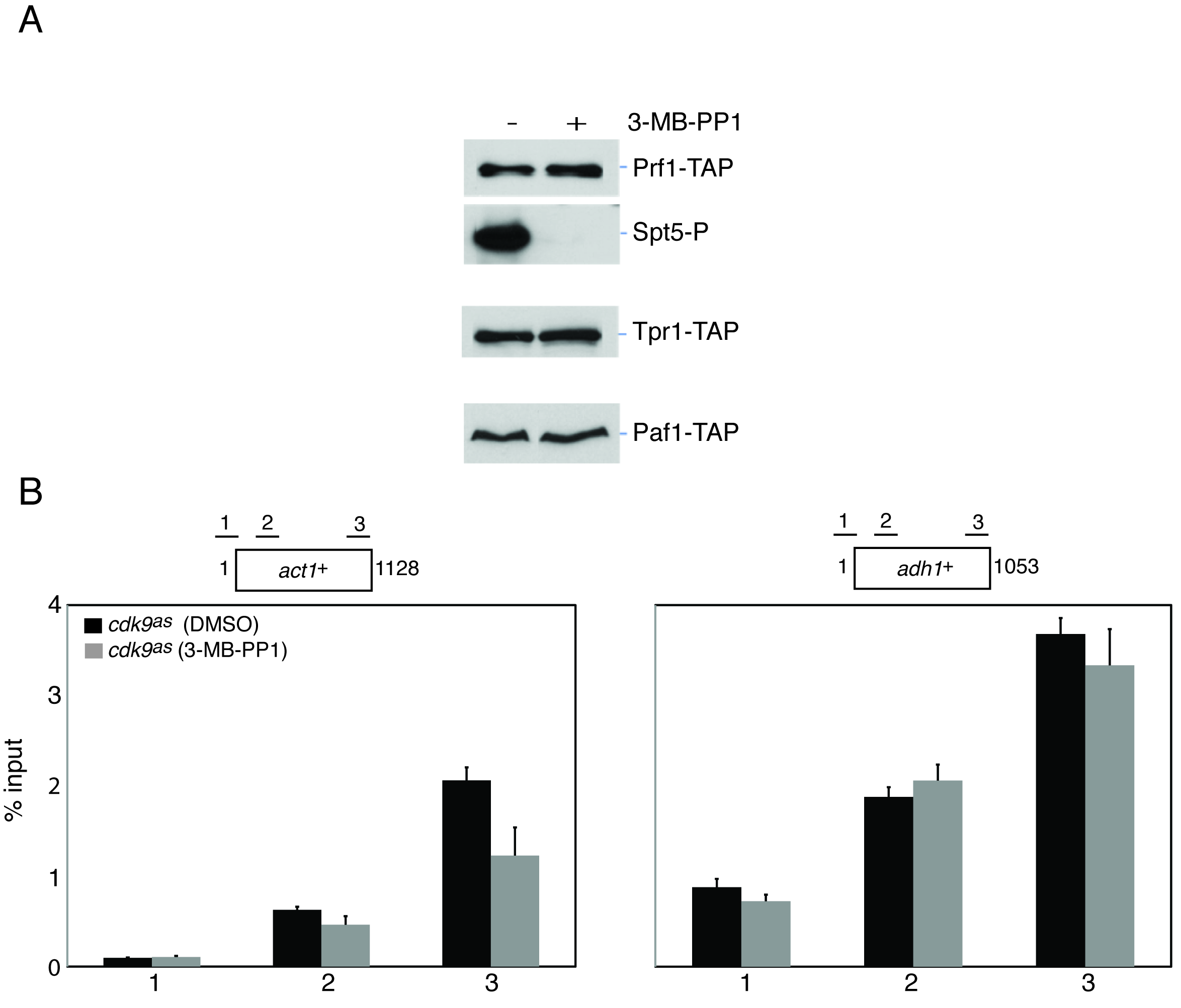

Supplement: Figure S3 — Inhibition of Cdk9 does not affect total levels of PAF or Prf1 and does not dramatically alter RNAPII occupancy at the constitutive act1 + and adh1 + genes. (A) The indicated TAP-tagged strains were treated with DMSO (−) or 20 µM 3-MB-PP1 (+) for 2 hours prior to whole-cell extract preparation and analysis by SDS-PAGE and western blotting. Levels of TAP-tagged proteins were assessed with the TAP antibody. (B) ChIP of RNAPII was carried out in the cdk9as strain and quantified by qPCR using primers specific to the nup189 + gene. Error bars denote standard deviations from three independent experiments. (TIF) [file pgen.1004029.s003.tif]

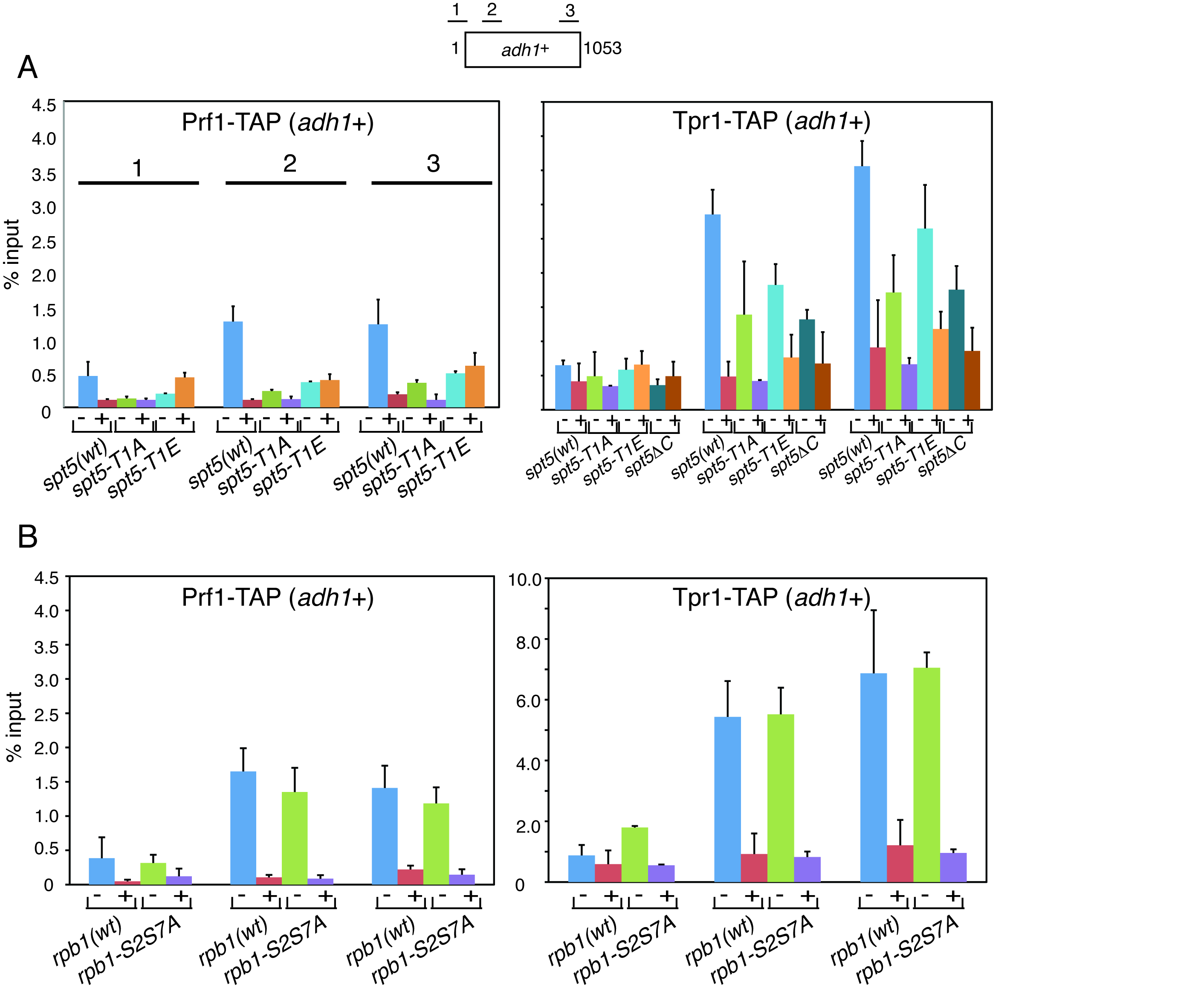

Supplement: Figure S4 — Prf1 and PAF are recruited to chromatin via alternate mechanisms. (A) and (B). Strains used for ChIP with IgG resin (recognizing the TAP tag) are indicated at the bottom. All strains also harbored the cdk9as allele and were treated with either DMSO (−) or 20 µM 3-MB-PP1 (+) for two hours before crosslinking for ChIP. Assays were quantified by qPCR using primers specific for the adh1 + gene. Length of the gene coding region (in base pairs) and positions of PCR amplicons are as indicated at the top. Error bars denote standard deviations from 2–3 independent experiments. (TIF) [file pgen.1004029.s004.tif]

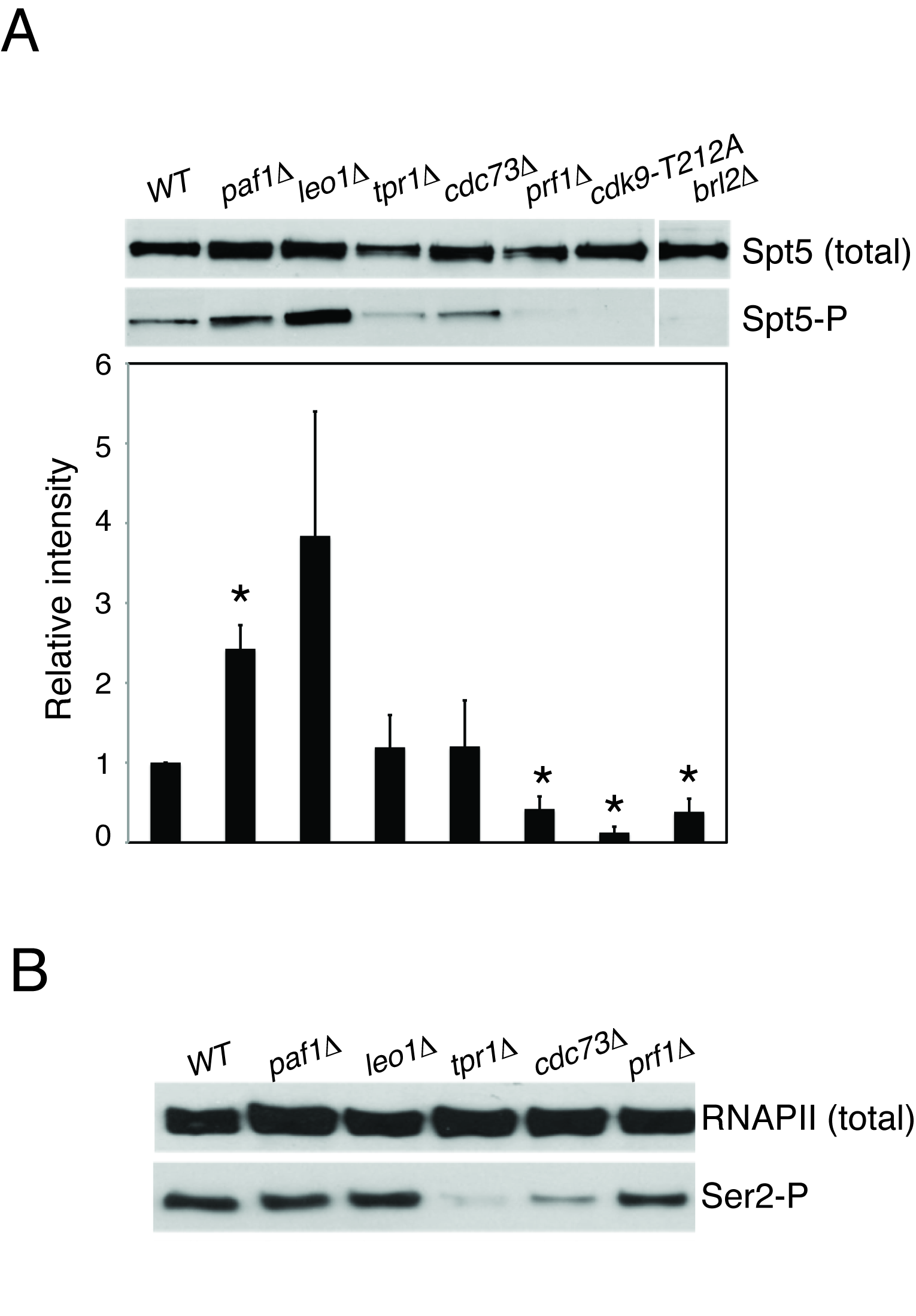

Supplement: Figure S5 — Prf1 and PAF differentially regulate phosphorylation of Spt5 CTD and Rpb1-Ser2. (A) Whole-cell extracts from strains of the indicated genotypes were analyzed by SDS-PAGE and western blotting with either the 8WG16 antibody (to detect total RNAPII) or the H5 antibody (to detect RNAPII phosphorylated at Rbp1-Ser2). Relative band intensities were quantified using ImageJ software and the ratios of Spt5-P signal to total Spt5 signal for each strain are plotted. Error bars denote standard deviation from 3 independent experiments and asterisks denote significant differences from the wild-type intensity (p<0.05; unpaired t-test). (B) As in (A). (TIF) [file pgen.1004029.s005.tif]

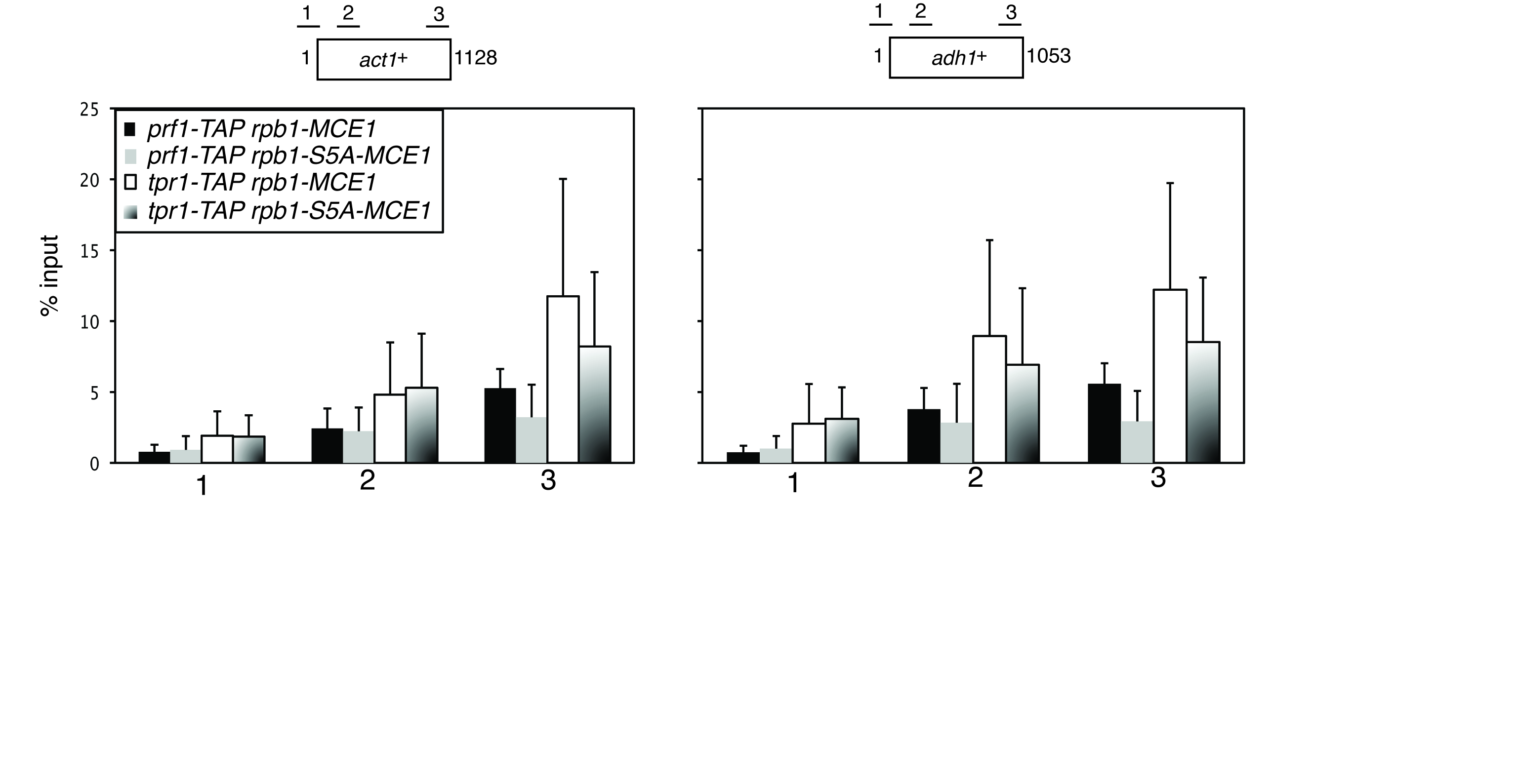

Supplement: Figure S6 — Rpb1-Ser5 is not required for recruitment of Prf1 or PAF to chromatin. ChIP was performed on the indicated strains with IgG resin (recognizing the TAP tag). Assays were quantified by qPCR using primers specific for the act1 + (left) or adh1 + (right) genes. Lengths of the gene coding regions (in base pairs) and positions of PCR amplicons are indicated at the top. Error bars denote standard deviations from 2–3 independent experiments. (TIF) [file pgen.1004029.s006.tif]

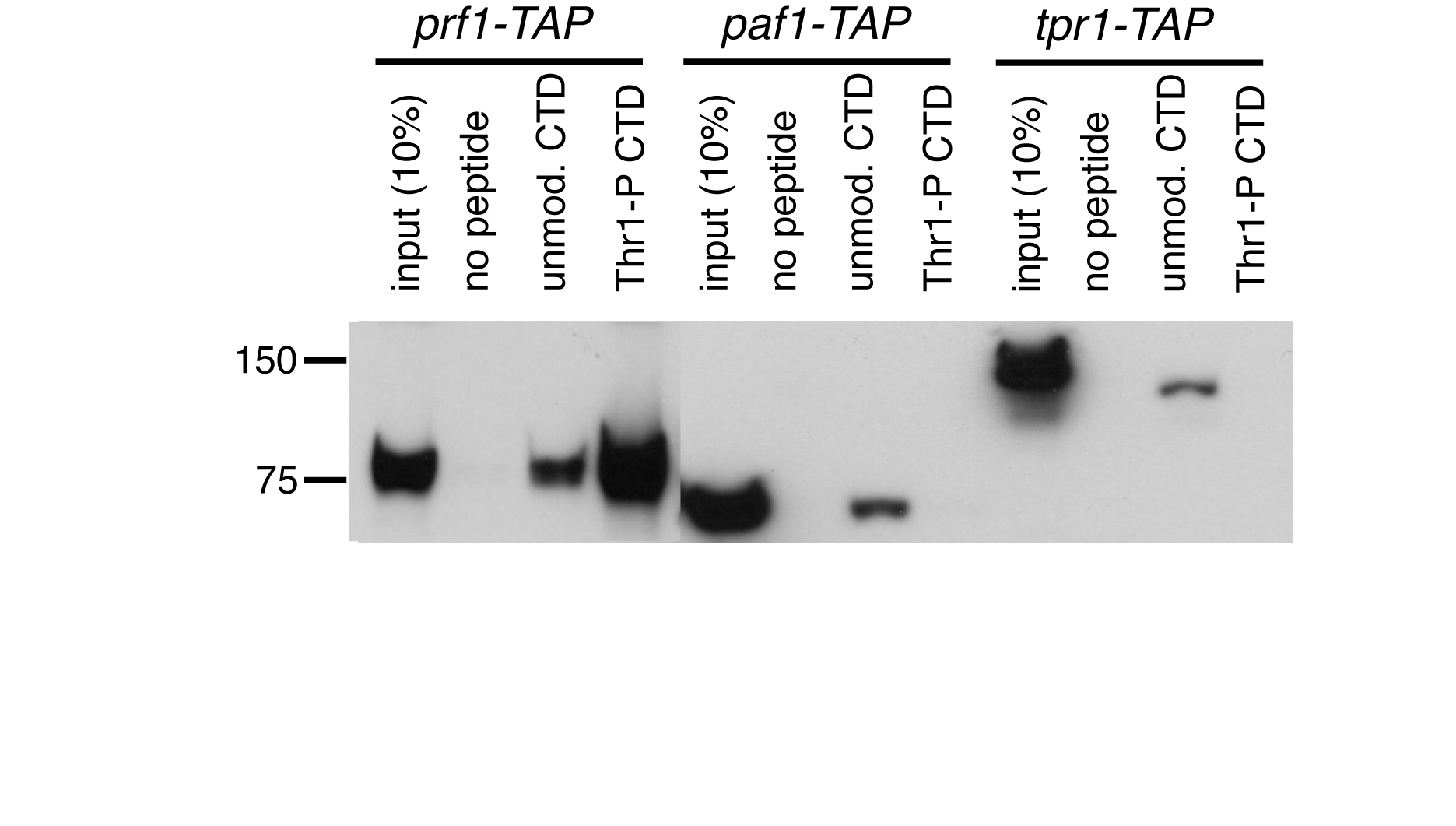

Supplement: Figure S7 — Purified Prf1 and PAF complex interact differently with the Spt5 CTD. Material purified from the TAP-tagged strains indicated at the top was incubated with magnetic steptavidin beads alone (“no peptide”) or beads coupled to the indicated biotinylated peptides. Input and bound fractions (50%) were analyzed by SDS-PAGE and western blotting with the TAP tag antibody. Molecular weight markers (in kD) are shown on the left. (TIF) [file pgen.1004029.s007.tif]

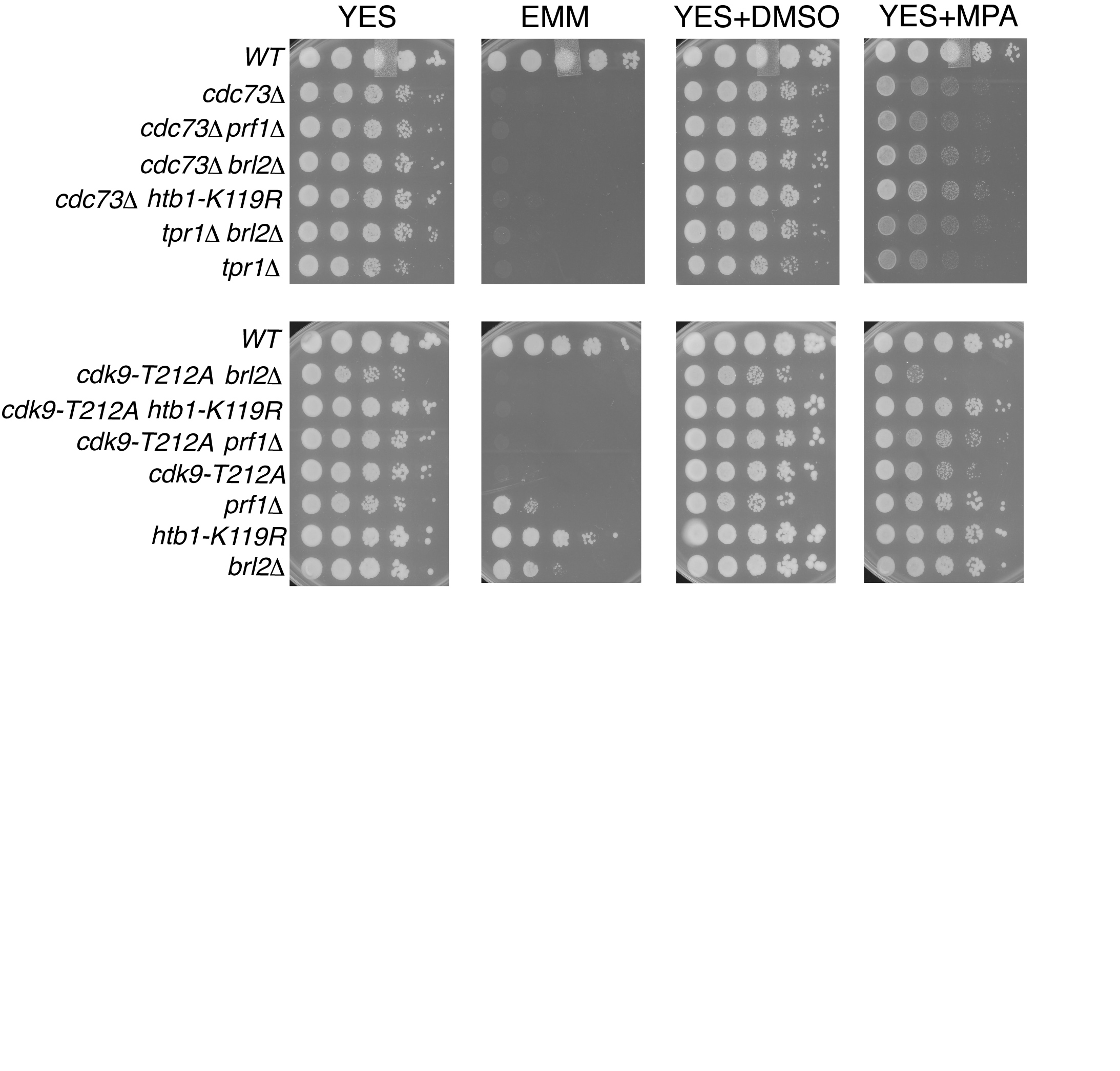

Supplement: Figure S8 — Phenotypes of cdk9 and PAF mutants do not require Prf1, Brl2, or H2Bub1. Five-fold serial dilutions of the indicated strains were spotted on agar plates containing rich media (YES), minimal media (EMM), rich media with dimethyl sulfoxide (YES+DMSO), or rich media with mycophenolic acid (YES+MPA). (TIF) [file pgen.1004029.s008.tif]

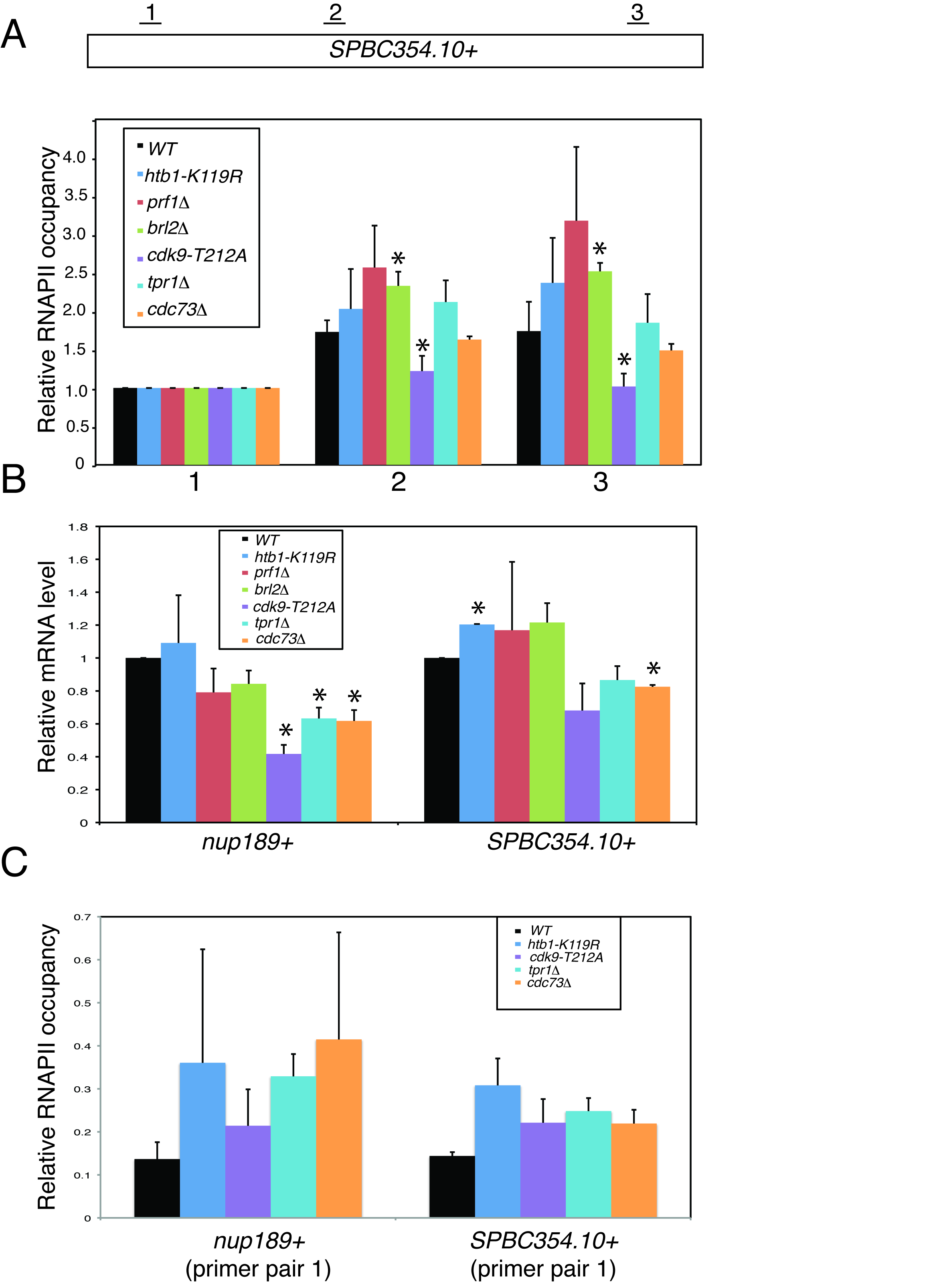

Supplement: Figure S9 — Differential effects of Prf1 and PAF pathway mutations on intragenic RNAPII distribution and gene expression. (A) ChIP of RNAPII was carried out in the indicated strains and quantified by qPCR using primers specific to the SPBC354.10 + gene. Values were normalized to that for primer pair 1. Error bars denote standard deviations from three independent experiments. Significant differences from wild-type values (unpaired t-test) are indicated. (B) Levels of mRNA from the indicated genes were quantified by qRT-PCR using primer pair 3 in either nup189 + or SPBC354.10 + and normalized to the signal for primer pair 3 in act1 +. The resulting value in the wild-type strain was set to 1. Error bars denote standard deviations from two independent experiments. Significant differences from wild-type values (p<0.05; unpaired t-test) are indicated by asterisks. (C) ChIP of RNAPII was carried out in the indicated strains and quantified by qPCR using primer pair 1 in either nup189 + or SPBC354.10 +. Values were normalized to that for primer pair 3 in act1 +. Error bars denote standard deviations from three independent experiments. Significant differences from wild-type values (p<0.05; unpaired t-test) are indicated by asterisks. (TIF) [file pgen.1004029.s009.tif]
