# Supplementary material for: The PAF Complex and Prf1/Rtf1 Delineate Distinct Cdk9-Dependent Pathways Regulating Transcription Elongation in Fission Yeast
Source: PLoS Genet. 2013 Dec 26;9(12):e1004029. doi: 10.1371/journal.pgen.1004029 (PMC3873232; doi:10.1371/journal.pgen.1004029)
Supplement: Table S1 — Mass spectrometry analysis of Tpr1-TAP and Prf1-TAP purifications. Shown are all proteins identified with a confidence level of 95% or greater in at least one of the samples analyzed. PAF complex components and Prf1 are highlighted in yellow. (PDF) [file pgen.1004029.s010.pdf]

Table S1. Mass spectrometry analysis of Prf1-TAP and Tpr1-TAP purifications.

#	Identified Proteins (78)	Accession Number	Molecular Weight	Percent Identification Probability no tag 1	Prf1-TAP 2	Tpr1-TAP 3	
1	SPAC15A10.15 [Schizosaccharomyces pombe]	gil2239191	72 kDa	95%	96%	100%	
2	Tpr1 [Schizosaccharomyces pombe]	gil2896142	119 kDa	0	0	100%	Tpr1
3	poly(A)+ RNA transport protein Ptr3p [imported] - fission yeast (Schizosaccharomyces pombe)	gil25293654 (+1)	113 kDa	59%	99%	100%	
4	hypothetical protein SPBC13E7.08c [Schizosaccharomyces pombe 972h-]	gil19113055	49 kDa	0	0	100%	Leo1
5	SPAC23G3.02c [Schizosaccharomyces pombe]	gil6912019	560 kDa	0	100%	99%	
6	SPAC664.03 [Schizosaccharomyces pombe]	gil6692010	51 kDa	75%	0	100%	Paf1
7	SPBC21D10.09c [Schizosaccharomyces pombe]	gil3560212	184 kDa	99%	98%	65%	
8	SPCC320.10 [Schizosaccharomyces pombe]	gil2995372	63 kDa	98%	95%	98%	
9	SPCC1259.08 [Schizosaccharomyces pombe]	gil4049541	43 kDa	98%	76%	0	
10	similar to Saccharomyces cerevisiae P9642.2 gene product, GENBANK Accession Number U40828 [Schizosaccharomyces pombe]	gil1749442 (+1)	55 kDa	97%	80%	0	
11	SPCC126.14 [Schizosaccharomyces pombe]	gil4008562	40 kDa	88%	98%	96%	
12	SPBC31E1.01c [Schizosaccharomyces pombe]	gil4494107 (+1)	179 kDa	97%	96%	88%	

13	SPBC1347.01c [Schizosaccharomyces pombe]	gil4456816	107 kDa	100%	84%	86%	
14	Mcs4 protein [Schizosaccharomyces pombe]	gil2065432	57 kDa	100%	86%	59%	
15	SPAC31A2.16 [Schizosaccharomyces pombe]	gil914894	127 kDa	67%	95%	96%	
16	SPAC2F3.11 [Schizosaccharomyces pombe]	gil2408065	43 kDa	0	0	96%	
17	SPCC1183.07 [Schizosaccharomyces pombe]	gil3650378	188 kDa	100%	82%	0	
18	SPAP8A3.05 [Schizosaccharomyces pombe]	gil5834790	78 kDa	0	100%	97%	
19	SPAC630.02 [Schizosaccharomyces pombe]	gil5734463	102 kDa	99%	91%	99%	
20	SPCP31B10.05 [Schizosaccharomyces pombe]	gil6066756	62 kDa	99%	85%	0	
21	SPAC3F10.16c [Schizosaccharomyces pombe]	gil1182063	70 kDa	98%	61%	86%	
22	SPBC17G9.02c [Schizosaccharomyces pombe]	gil5738523	43 kDa	0	0	100%	Cdc73
23	SPBC1861.08c [Schizosaccharomyces pombe]	gil5734581	27 kDa	85%	92%	100%	
24	SPAC23D3.13c [Schizosaccharomyces pombe]	gil1039351	181 kDa	99%	0	45%	
25	Moc2 RNA helicase [Schizosaccharomyces pombe]	gil2992158 (+1)	70 kDa	98%	0	97%	
26	rpl13 [Schizosaccharomyces pombe]	gil6692012	24 kDa	95%	73%	97%	
27	SPAC2F3.10 [Schizosaccharomyces pombe]	gil2408064	108 kDa	96%	67%	76%	
28	SPCC794.08 [Schizosaccharomyces pombe]	gil3150121	90 kDa	96%	75%	76%	
29	cox1I1a maturase [Schizosaccharomyces pombe]	gil6468460	90 kDa	95%	46%	46%	
30	SPBC211.03c [Schizosaccharomyces	gil6983767	165 kDa	100%	71%	0	

	pombe]						
31	SPAC6C3.04 [Schizosaccharomyces pombe]	gil4582397	53 kDa	99%	0	0	
32	ORF N313 [Schizosaccharomyces pombe]	gil1507668 (+1)	34 kDa	59%	99%	91%	
33	SPACUNK4.12c [Schizosaccharomyces pombe]	gil3395558	112 kDa	90%	0	99%	
34	SPBC56F2.04 [Schizosaccharomyces pombe]	gil3116134	285 kDa	99%	0	0	
35	SPAC3G6.11 [Schizosaccharomyces pombe]	gil2408082	96 kDa	0	0	99%	
36	sdh2 [Schizosaccharomyces pombe]	gil7523471	29 kDa	0	98%	99%	
37	SPBC887.11 [Schizosaccharomyces pombe]	gil3850107	52 kDa	99%	50%	0	
38	SPBC2G2.07c [Schizosaccharomyces pombe]	gil2956774	26 kDa	0	99%	97%	
39	hypothetical protein SPBC146.01 [Schizosaccharomyces pombe 972h-]	gil63054716 (+1)	118 kDa	54%	70%	98%	
40	SPAC212.11 [Schizosaccharomyces pombe]	gil9967692	216 kDa	96%	0	98%	
41	SPBC2G5.08 [Schizosaccharomyces pombe]	gil3850073	66 kDa	84%	70%	96%	
42	SPCC965.01 [Schizosaccharomyces pombe]	gil3395592	20 kDa	95%	0	54%	
43	SPBC651.09c [Schizosaccharomyces pombe]	gil4467281	64 kDa	0	100%	0	Prf1
44	SPCC970.09 [Schizosaccharomyces pombe]	gil3560234	125 kDa	100%	0	0	
45	SPBC215.08c [Schizosaccharomyces pombe]	gil3873545	127 kDa	100%	0	0	
46	SPCC830.01c [Schizosaccharomyces pombe]	gil5738867 (+1)	144 kDa	99%	0	0	
47	rad54 [Schizosaccharomyces pombe]	gil2239180 (+1)	97 kDa	0	99%	0	

48	myosin-II; Myp2p [Schizosaccharomyces pombe]	gil2731818 (+2)	243 kDa	99%	54%	0
49	SPAC20G8.08c [Schizosaccharomyces pombe]	gil2094863	108 kDa	99%	73%	0
50	acetolactate synthase	gil406192 (+1)	73 kDa	98%	0	0
51	probable pseudogene, homologous to N terminal of transmembrane channel [imported] - fission yeast (Schizosaccharomyces pombe)	gil11359266	19 kDa	98%	0	85%
52	kinesin-related protein cut7 - fission yeast (Schizosaccharomyces pombe)	gil1076897 (+3)	121 kDa	98%	0	0
53	SPAPJ696.01c [Schizosaccharomyces pombe]	gil6562184	61 kDa	97%	0	0
54	hypothetical protein SPAC32A11.02c [Schizosaccharomyces pombe 972h-]	gil19114687	97 kDa	0	97%	65%
55	SPBC19C7.08c [Schizosaccharomyces pombe]	gil3218412	78 kDa	97%	0	0
56	SPAC57A10.03 [Schizosaccharomyces pombe]	gil2058370	17 kDa	96%	89%	0
57	SPAC890.06 [Schizosaccharomyces pombe]	gil6594230	148 kDa	55%	96%	0
58	SPAC4G9.08c [Schizosaccharomyces pombe]	gil1204209	130 kDa	96%	70%	0
59	SPCC285.05 [Schizosaccharomyces pombe]	gil3581905	38 kDa	95%	86%	0
60	hypothetical protein SPAC31G5.19 [Schizosaccharomyces pombe 972h-]	gil19114932	135 kDa	0	95%	74%
61	actin [Schizosaccharomyces pombe]	gil1304269 (+1)	42 kDa	100%	0	0
62	hypothetical protein SPCC777.16c [Schizosaccharomyces pombe 972h-]	gil63054446 (+2)	95 kDa	99%	0	0

63	SPAC11G7.04 [Schizosaccharomyces pombe]	gil2408009 (+3)	15 kDa	99%	0	0
64	SPAC1F3.06c [Schizosaccharomyces pombe]	gil1256517	223 kDa	98%	0	0
65	Chain B, Crystal Structure Of A Protein With Similarity To Flavin- Containing Monooxygenases And To Mammalian Dimethylalanine Monooxygenases	gil60593736	51 kDa	98%	0	0
66	SEC61 protein [Schizosaccharomyces pombe]	gil1870152	53 kDa	97%	0	0
67	SPAC2G11.10c [Schizosaccharomyces pombe]	gil1019408	44 kDa	0	0	97%
68	SPBC1604.20c [Schizosaccharomyces pombe]	gil4376084 (+1)	70 kDa	97%	0	0
69	SPCC14G10.02 [Schizosaccharomyces pombe]	gil3560186 (+1)	179 kDa	95%	0	0
70	fdh [Schizosaccharomyces pombe]	gil14715439 (+1)	100 kDa	98%	0	0
71	SPAC1039.05c [Schizosaccharomyces pombe]	gil6594264	89 kDa	0	98%	0
72	ribosomal protein S9 [Schizosaccharomyces pombe]	gil2897739 (+3)	21 kDa	0	0	97%
73	tup1 [Schizosaccharomyces pombe]	gil5734475 (+1)	64 kDa	96%	0	0
74	ribosomal protein S31 [Schizosaccharomyces pombe]	gil1783177 (+3)	10 kDa	96%	0	0
75	SPAC1A6.08c [Schizosaccharomyces pombe]	gil2414591	33 kDa	95%	0	0
76	SPBC359.05 [Schizosaccharomyces pombe]	gil7838270	167 kDa	0	0	95%
77	SPAC29A4.14c [Schizosaccharomyces pombe]	gil23095751 (+1)	39 kDa	95%	0	0
78	aro1 [Schizosaccharomyces pombe]	gil7019762	174 kDa	95%	0	0
