# Supplementary material for: The PAF Complex and Prf1/Rtf1 Delineate Distinct Cdk9-Dependent Pathways Regulating Transcription Elongation in Fission Yeast
Source: PLoS Genet. 2013 Dec 26;9(12):e1004029. doi: 10.1371/journal.pgen.1004029 (PMC3873232; doi:10.1371/journal.pgen.1004029)
Supplement: Table S2 — S. pombe strains used in this study. (PDF) [file pgen.1004029.s011.pdf]

Table S2. *S. pombe* strains used in this study.

Name	Genotype	Source
JTB204	*h- ade6-M216*	Tanny et al 2007
JTB483	*h- paf1-TAP::kanMX6 ade6-M216*	This study
JTB435	*h- tpr1-TAP::kanMX6 ade6-M216*	This study
JTB202	*h- prf1-TAP::kanMX6 ade6-M216*	This study
JTB86	*h+ htb1-K119R::kanMX6 ade6-M216*	Tanny et al 2007
JTB409	*h+ paf1Δ::kanMX4 ade6-M216 ura4 D18 leu1-32*	Bioneer
JTB410	*h+ leo1Δ::kanMX4 ade6-M216 ura4 D18 leu1-32*	Bioneer
JTB411	*h+ tpr1Δ::kanMX4 ade6-M216 ura4 D18 leu1-32*	Bioneer
JTB412	*h+ cdc73Δ::kanMX4 ade6-M216 ura4 D18 leu1-32*	Bioneer
JTB113	*h- prf1Δ::hphMX4 ade6*	This study
JTB122	*h+ brl2Δ::hphMX4 ade6-M210*	Tanny et al 2007
JTB80-2	*h+ set1Δ::kanMX6 ade6*	Tanny et al 2007
JTB142	*h- set2Δ::hphMX6 ade6*	This study
JTB321	*h+ cdk9-T212A::kanMX6 leu1-32 ura4-D18 his3-D1 ade6-M210*	Sanso et al 2012
JTB392	*h? cdk9^{as}::KanMX6 prf1-TAP::kanMX6 ade6*	This study
JTB452	*h? tpr1-TAP::kanMX6 cdk9^{as}::natMX6 leu1-32? ura4-D18? his3-D1? ade6*	This study
JTB491	*h? paf1-TAP::kanMX6 cdk9^{as}::natMX6 leu1-32? ura4-D18? his3-D1? ade6*	This study
JTB489	*h? spt5(wt)::ura4+ cdk9^{as}::kanMX6 prf1-TAP::kanMX6 ade6 ura4? leu1? his3?*	This study
JTB490	*h? spt5-T1E::ura4+ cdk9^{as}::kanMX6 prf1-TAP::kanMX6 ade6 ura4? leu1? his3?*	This study
JTB494	*h? spt5-T1A::ura4+ cdk9^{as}::natMX6 prf1-TAP::kanMX6 leu1? ura4-D18 his3? ade6*	This study
JTB576	*h? cdk9^{as}::hphMX6 tpr1-TAP::kanMX6 spt5(wt)::ura4+ leu1-32 his3-D1 ade6-M210*	This study
JTB577	*h? cdk9^{as}::hphMX6 tpr1-TAP::kanMX6 spt5-T1A::ura4+ leu1-32 ura4-D18 his3-D1*	This study
JTB578	*h? cdk9^{as}::hphMX6 tpr1-TAP::kanMX6 spt5-T1E::ura4+ leu1-32 ura4-D18 his3-D1*	This study
JTB579	*h? cdk9^{as}::hphMX6 tpr1-TAP::kanMX6 spt5ΔC::ura4+ leu1-32 his3-D1 ade6*	This study
JTB591	*h? cdk9^{as}::hphMX6 prf1-TAP::kanMX6 rpb1(wt)::natMX6 leu1-32? ura4-D18? his3-D1? ade6*	This study
JTB592	*h? cdk9^{as}::hphMX6 prf1-TAP::kanMX6 rpb1-S2A-S7A::natMX6 leu1-32? ura4-D18? his3-D1? ade6*	This study
JTB570	*h? cdk9^{as}::hphMX6 tpr1-TAP::kanMX6 rpb1wt::natMX6 ura4-D18 his3-D1? ade6-M210? leu1-32?*	This study
JTB571	*h? cdk9^{as}::hphMX6 tpr1-TAP::kanMX6 rpb1-S2A-S7A::natMX6 ura4-D18 his3-D1? ade6-M210? leu1-32?*	This study
JTB612	*h? rpb1-MCE1::natMX6 prf1-TAP::kanMX6 ade6 leu1-32? ura4-D18?*	This study

JTB612	*h? rpb1-MCE1::natMX6 prf1-TAP::kanMX6 ade6 leu1-32? ura4-D18? his3-D1?*	This study
JTB613	*h? prf1-TAP::kanMX6 rpb1-S5A-MCE1::natMX6 ade6 leu1-32? ura4-D18? his3-D1?*	This study
JTB614	*h? tpr1-TAP::kanMX6 rpb1-S5A-MCE1::natMX6 leu1-32 his3-D1? ade6-M210?*	This study
JTB615	*h? tpr1-TAP::kanMX6 rpb1-S5A-MCE1::natMX6 leu1-32 his3-D1? ade6-M210?*	This study
JTB606	*h? cdc73Δ::kanMX4 rtf1Δ::hphMX4 ade6-M216 leu1-32*	This study
JTB607	*h? cdc73Δ::kanMX4 htb1-K119R::kanMX6 ade6-M216 leu1-32*	This study
JTB608	*h? cdc73Δ::kanMX4 brl2Δ::hphMX4 ade6 leu1-32?*	This study
JTB609	*h? tpr1Δ::kanMX4 brl2Δ::hphMX4 ade6 leu1-32?*	This study
JTB610	*h? cdk9-T212A::kanMX6 prf1Δ::hphMX4 ade6 ura4-D18 his3-D1?*	This study
JTB335	*h? cdk9-T212A::kanMX6 brl2Δ::hphMX4 leu1-32 ura4-D18 his3? ade6*	Sanso et al 2012
JTB429	*h? cdk9-T212A htb1-K119R leu1-32? ura4-D18? his3-D1? ade6*	This study
JTB386	*h? cdk9^{as}::KanMX6 brl2Δ::hphMX4 ade6 ura4-D18 his3-D1*	This study
JTB495	*h? prf1Δ::hphMX4 cdk9^{as}::natMX6 leu1-32? ura4-D18? his3-D1? ade6*	This study
JTB508	*h? cdk9^{as}::natMX6 htb1-K119R::kanMX6 ade6 leu1-32? ura4-D18? his3-D1?*	This study
JTB641	*h? prf1-TAP::kanMX6 tpr1-13xmyc::hphMX6*	This study
