# Supplementary material for: The PAF Complex and Prf1/Rtf1 Delineate Distinct Cdk9-Dependent Pathways Regulating Transcription Elongation in Fission Yeast
Source: PLoS Genet. 2013 Dec 26;9(12):e1004029. doi: 10.1371/journal.pgen.1004029 (PMC3873232; doi:10.1371/journal.pgen.1004029)
Supplement: Text S1 — Supplemental materials and methods. (PDF) [file pgen.1004029.s012.pdf]

Text S1. Supplemental Materials and methods.

Mbogning et al.

Supplemental Materials and Methods

Protein identification by mass spectrometry: Mass spectrometry analyses were carried out by the proteomics facility at the Institute for Research in Immunology and Cancer (Université de Montreal, Montreal, Canada). Coommassie-blue stained polypeptides were excised and the gel slices were destained, reduced in 10 mM DTT for 1 hour at 56ºC, and alkylated in 55 mM chloroacetamide for one hour at room temperature. After washing in 50 mM ammonium bicarbonate, the gel pieces were shrunk in 100% acetonitrile (ACN). Proteolysis was performed using trypsin in 50 mM ammonium bicarbonate for 8 hours at 37ºC. Reactions were subjected to continuous mixing in a Thermomixer (Eppendorf) at 600 rpm. The peptides were finally extracted in 90% ACN/0.5M urea and dried in a speed vac. Samples in solution were dried in a speedvac and resuspended in 50 μl of 50 mM ammonium bicarbonate. TCEP (tris(2-carboxyethyl)phosphine) was added to a concentration of 5mM. Reactions were subjected to continuous mixing in a Thermomixer (Eppendorf) at 600 rpm. Chloroacetamide (30 μl) at 55 mM was added and the samples were again incubated in a Thermomixer for 30 min. Trypsin (1μg) was added and the samples were digested overnight at 37 °C. Samples were then dried in a speed vac.

Samples were resolubilized in 5% ACN/2% formic acid (FA). Samples were separated on a home-made C_{18} column (150 μm × 10 cm) using an Eksigent nanoLC-2D system. A 56-min gradient from 5–60% acetonitrile (0.2% FA) was used to elute peptides from a homemade reversed-phase column (150 μm i.d. x 100 mm) with a flow rate set at 600 nl/min. The column was directly connected to a nanoprobe interfaced with an LTQ-Orbitrap Elite mass spectrometer from Thermo-Fisher. Each full MS spectrum was followed by 12 MS/MS spectra (thirteen scan events), where the twelve most abundant multiply charged ions were selected for MS/MS sequencing. Tandem MS experiments were performed using collision-induced dissociation in the linear ion trap. The data were processed using the 2.4 Mascot (Matrix Science) search engine with tolerance parameters

set to 15 ppm and 0.5 Da for the precursor and the fragment ions respectively to achieve a false-positive rate of less than 1% ($p < 0.01$). The selected variable modifications were carbamidomethyl (C), deamidation (NQ), oxidation (M) and phosphorylation (STY). Tandem mass spectra were blasted against the Uniprot *S. pombe* database. Identity of polypeptides was assigned based on a normalized spectrum count of >40.

RNA isolation and qRT-PCR. Quantification of mRNA levels was carried out as described previously (reference 11).
